# The Metabolic Rheostat Index Uncovers Pathway-Level Coordination, Immunosuppressive Microenvironment Remodeling, and Therapeutic Co-Vulnerabilities in Pancreatic Ductal Adenocarcinoma

**DOI:** 10.3390/cimb48080820

**Published:** 2026-08-12

**Authors:** Funda Demirtas Korkmaz, Yasemin Sevim, Zekeriya Duzgun, Asuman Deveci Ozkan

**Affiliations:** 1 Department of Medical Biology, Faculty of Medicine, Giresun University, 28200 Giresun, Türkiye; yasemin.sevim@giresun.edu.tr (Y.S.);; 2 Department of Medical Biology, Faculty of Medicine, Sakarya University, 54290 Sakarya, Türkiye

**Keywords:** pancreatic ductal adenocarcinoma, single-cell RNA sequencing, metabolic reprogramming, Metabolic Rheostat Index, glycolysis, oxidative phosphorylation, tumor microenvironment, metabolic plasticity

## Abstract

Pancreatic ductal adenocarcinoma (PDAC) is characterized by extensive metabolic reprogramming, yet how major metabolic pathways are organized at single-cell resolution, and how this relates to the tumor microenvironment and therapeutic vulnerabilities, remains incompletely understood. We analyzed 32,227 cells from the GSE155698 discovery cohort and 32,627 cells from an independent healthy-pancreas reference (GSE229413; *n* = 5 donors) and introduce the Metabolic Rheostat Index (MRI), the standard deviation across per-cell glycolysis, OXPHOS, and hypoxia pathway scores. A pseudobulk analysis, hallmark gene-set validation, unified-threshold classification, a GDSC2 drug-sensitivity analysis with robust regression, EPIC tumor-purity-adjusted deconvolution, and a survival analysis of 183 TCGA-PAAD samples were performed. The pseudobulk analysis revealed a monotonic MRI gradient from healthy pancreata (mean = 0.259) to adjacent-normal tissue (0.322) to PDAC tumor tissue (0.352; *p* = 0.0039, Cohen’s d = 1.437), indicating progressive metabolic polarization during malignant transformation driven predominantly by cancer-associated fibroblasts rather than malignant epithelial cells. The glycolysis–hypoxia correlation was stronger in tumor cells (r = 0.121 vs. 0.019), and ERK/MAPK inhibitors showed the greatest drug sensitivity (r = 0.597, *p* = 0.0008). The Warburg Index correlated with fibroblast infiltration (partial r = 0.312, *p* = 1.76 × 10^−5^) but was not an independent prognostic marker (HR = 0.78, *p* = 0.321). These findings indicate that PDAC tissue undergoes progressive, stroma-driven metabolic polarization associated with an immunosuppressive microenvironment, providing a multi-dimensional single-cell framework for future metabolic-targeted therapeutic strategies.

## 1. Introduction

Pancreatic ductal adenocarcinoma (PDAC) remains one of the most lethal solid malignancies, with an overall five-year survival rate of approximately 10–12%, and patients with advanced disease continue to have a poor prognosis despite recent therapeutic advances [1]. This poor clinical outcome is largely driven by late-stage diagnosis, a dense desmoplastic tumor microenvironment (TME) that creates physical and biochemical barriers to drug delivery, strong resistance to conventional chemotherapeutic agents, and significant intra-tumoral heterogeneity [2,3,4].

Among the hallmarks of cancer, metabolic reprogramming has emerged as a key feature contributing to tumor progression and a potential therapeutic target [5]. The Warburg effect, whereby cancer cells preferentially generate ATP through aerobic glycolysis rather than oxidative phosphorylation (OXPHOS), even under oxygen-sufficient conditions, represents one of the earliest and most well-characterized metabolic alterations in PDAC [6]. However, this glycolysis-centered model has since been refined: PDAC cells exhibit substantial metabolic flexibility, utilizing alternative substrates, including glutamine, fatty acids, and stromal-derived metabolites [7,8]. The Reverse Warburg Effect further highlights the metabolic interplay between cancer cells and stromal components [9]. Collectively, these findings have led to an expanded framework of cancer metabolism, encompassing deregulated nutrient uptake, adaptive metabolic reprogramming, and the redirection of intermediates toward anabolic processes [10].

Within PDAC, hypoxia is a major microenvironmental driver of metabolic state. Sustained activation of HIF-1α promotes glycolysis and hypoxia-response pathways [11], while oncogenic KRAS, which is mutated in over 90% of PDAC cases, further enhances glycolytic flux through interactions with key metabolic enzymes, including hexokinase 1 [12]. Subcellular localization of phosphoglycerate kinase 1 (PGK1) has been shown to influence the balance between proliferative glycolytic and invasive OXPHOS-dependent phenotypes, contributing to metabolic heterogeneity within tumor cell populations [13]. Importantly, this metabolic heterogeneity is not merely a consequence of oncogenic signaling but is actively shaped by microenvironmental cues: matrix stiffness drives HIF-1α activation through a CLIC1-ROS signaling axis [11], and microenvironmental signals rather than cell-intrinsic genetic programs predominantly determine the transcriptional and metabolic state of PDAC cells [14].

Recent advances in single-cell RNA sequencing (scRNA-seq) have enabled the characterization of metabolic gene expression at single-cell resolution, revealing heterogeneity that is not captured by bulk transcriptomic analyses [15]. Pan-cancer and PDAC-specific studies have demonstrated consistent upregulation of glycolysis alongside variable mitochondrial activity, as well as spatially resolved metabolic diversity associated with clinical outcomes [16,17]. Moreover, transcriptomic and single-cell analyses have revealed a close interplay between metabolic reprogramming and the stromal and immune composition of the PDAC tumor microenvironment, with enhanced glycolytic activity and lactate metabolism contributing to CAF-mediated immunosuppressive phenotypes and reduced cytotoxic immune activity [18]. Despite these advances, most current approaches evaluate metabolic pathways independently, leaving their coordinated regulation at the single-cell level insufficiently understood. Furthermore, the relationship between single-cell metabolic architecture, bulk tumor microenvironment composition, and pharmacological vulnerability has not been systematically characterized in PDAC.

To address these limitations, we introduce the Metabolic Rheostat Index (MRI), a single-cell metric that quantifies inter-pathway metabolic divergence by calculating the standard deviation across glycolysis, OXPHOS, and hypoxia pathway activities within individual cells. Unlike measures of metabolic intensity, MRI captures the relative balance between pathways: high MRI values indicate dominance of a single metabolic program, whereas low values reflect coordinated pathway activity. We apply this framework to a publicly available scRNA-seq dataset (GSE155698; 32,227 cells from PDAC and normal pancreas; Steele et al., 2020 [19] and independently validate all primary findings in a second PDAC cohort (GSE205013; 34,000 cells from 17 treatment-naive primary tumors; Werba et al., 2023 [20]) and against a healthy pancreas reference dataset (GSE229413; 32,627 cells from 5 healthy donors). To address pseudoreplication concerns, all primary comparisons are performed at the sample level using pseudobulk aggregation. We further validate pathway scoring using MSigDB hallmark gene sets and UCell rank-based scoring, apply unified global threshold classification for metabolic state assignment, characterize drug sensitivity profiles in 30 PDAC cell lines using GDSC2 data with robust regression, and apply EPIC deconvolution with tumor purity-adjusted partial correlation analysis to 183 TCGA-PAAD samples. We construct a protein–protein interaction network to provide mechanistic context for the observed inter-pathway correlation patterns. Our results show that PDAC tumor tissue exhibits increased inter-pathway divergence compared to healthy pancreata, following a monotonic gradient from healthy normal to adjacent normal to tumor confirmed at the sample level with a large effect size (Cohen’s d = 1.437), driven predominantly by cancer-associated fibroblasts and stromal/compositional remodeling rather than by malignant epithelial cells themselves, in part via HIF-1α-mediated coupling of glycolysis and hypoxia programs, and that a high Warburg Index associates with an immunosuppressive microenvironment and ERK–metabolic co-vulnerabilities, providing a multi-dimensional translational framework for PDAC metabolic characterization.

## 2. Materials and Methods

### 2.1. scRNA-Seq Discovery Dataset

Publicly available single-cell RNA sequencing data were obtained from the Gene Expression Omnibus (GEO) repository under accession number GSE155698 (Steele et al., 2020, Nature Cancer [19]). This dataset comprises 10× Genomics Chromium (10× Genomics, Pleasanton, CA, USA) single-cell transcriptional profiles of cells isolated from 16 primary pancreatic ductal adenocarcinoma tissue samples and 3 matched adjacent normal pancreatic tissue samples. After quality control filtering, which excluded cells with fewer than 100 detected genes, more than 8000 detected genes, or more than 40% mitochondrial RNA content, a total of 32,227 cells, were retained for downstream analysis, comprising 24,515 tumor-derived and 7712 normal pancreatic cells. All data were obtained from a publicly available de-identified repository; no additional institutional review board approval was required.

### 2.2. Healthy Normal Pancreas Reference Dataset

To establish an independent healthy pancreas reference with a sufficient sample size for the pseudobulk analysis, single-cell RNA sequencing data were obtained from GEO accession GSE229413 [21], comprising 5 healthy human donors profiled using 10× Genomics. After quality control filtering (nFeature_RNA > 100, nFeature_RNA < 8000, percent.mt < 40%), 32,627 cells were retained. The same normalization, pathway scoring, and MRI calculation pipeline were applied identically to the discovery cohort.

### 2.3. Dimensionality Reduction and Clustering

Counts were log-normalized using a scale factor of 10,000. Approximately 2000 highly variable genes were identified using the variance-stabilizing transformation method. Data were scaled, and principal components were computed (RunPCA, npcs = 30). Unsupervised graph-based clustering was performed on the PCA embedding (FindNeighbors, dims = 1–20; FindClusters, Louvain algorithm, resolution = 0.8), resolving 37 transcriptionally distinct clusters. Uniform Manifold Approximation and Projection (UMAP) embeddings were computed from PCA space (dims = 1–20) for visualization. Each cluster was assigned to a major cell lineage using a curated panel of canonical marker genes visualized as a dot plot. The lineages identified included malignant epithelial cells (KRT19, EPCAM, and MUC1), ductal epithelial cells (KRT7, KRT18, and TFF1), cancer-associated fibroblasts (FAP, COL1A1, and ACTA2), macrophages/myeloid cells (CD68, CD163, and CSF1R), cytotoxic T cells (CD8A and GZMB), T helper cells (CD3D and CD4), regulatory T cells (FOXP3), B cells (MS4A1), NK cells (NCAM1 and NKG7), endothelial cells (VWF), acinar cells (PRSS1 and CTRB1), and stellate cells (RGS5 and PDGFRB). Malignant epithelial cell identity was assigned based on canonical marker expression (KRT19, EPCAM, and MUC1) rather than copy-number-variation analysis; cells from adjacent normal tissue meeting these marker criteria are therefore referred to as epithelial/ductal-like rather than confirmed malignant throughout, as CNV-based validation of malignancy was not performed in the present study. All analyses were performed using Seurat v5.3.0 in R v4.4.1.

### 2.4. Metabolic Pathway Scoring

Three metabolic programs were scored per cell using compact five-gene sets: glycolysis (LDHA, PKM, ENO1, GAPDH, and SLC2A1), oxidative phosphorylation/OXPHOS (COX5A, UQCRC1, ATP5F1A, SDHB, and NDUFB8), and hypoxia response (HIF1A, VEGFA, CA9, PDK1, and BNIP3). Pathway scores were computed using Seurat::AddModuleScore with cohort-specific background gene sets, which normalizes each score against background gene sets sampled from the same expression-level bins, providing an implicit per-cell normalization robust to between-sample library-size differences. GSE155698 was generated using a unified 10× Genomics pipeline with consistent cell isolation and library preparation protocols across all 19 samples, and the same scoring pipeline was applied independently to GSE229413; formal batch correction (e.g., Harmony or ComBat-seq) integrating the two datasets was not applied. This choice, and its implications for the cross-dataset pseudobulk comparison, are discussed as a limitation below. To evaluate robustness to gene-set composition, all three pathways were independently rescored using extended sets of 14–15 genes per pathway (Supplementary Figure S4). In addition, pathway scoring was validated using: (1) MSigDB hallmark gene sets (glycolysis: 200 genes; OXPHOS: 201 genes; hypoxia: 200 genes) with AddModuleScore; and (2) UCell rank-based scoring (v2.0) with both compact and hallmark gene sets. UCell scoring is based on the Mann–Whitney U statistic and is robust to library size variation. Concordance between AddModuleScore–compact and UCell–compact was assessed by Pearson correlation per pathway (glycolysis r = 0.859; OXPHOS r = 0.778; hypoxia r = 0.830).

### 2.5. Metabolic Rheostat Index

The Metabolic Rheostat Index (MRI) was defined as the standard deviation (SD) across the three per-cell pathway scores (glycolysis, OXPHOS, hypoxia) for each individual cell. A high MRI value indicates strong inter-pathway divergence (metabolic polarization); a low MRI value indicates uniform cross-pathway engagement (metabolic uniformity). MRI robustness was evaluated using the coefficient of variation (CV) and Shannon entropy as alternative dispersion metrics. Shannon entropy was computed as H = −Σp_i log(p_i), where pathway score vectors were transformed into a non-negative probability distribution by subtracting the minimum value and normalizing to sum to unity. Because Shannon entropy increases with pathway uniformity, entropy-based MRI was defined as MRI_entropy = 1 − H_normalized, such that higher values indicate greater inter-pathway divergence. CV-based MRI yielded a non-significant result (*p* = 0.23), attributable to mathematical instability of ratio-based dispersion measures when mean pathway scores approach zero. Shannon entropy-based MRI confirmed the SD-based direction (*p*.adj = 2.1 × 10^−13^; Supplementary Figure S3).

### 2.6. Pseudobulk Sample-Level Analysis

To address pseudoreplication concerns arising from treating individual cells as independent observations, all primary MRI comparisons were repeated at the sample level using pseudobulk aggregation. Per-cell MRI values were aggregated to per-sample means, yielding one observation per patient or donor. Sample-level comparisons between PDAC tumor (*n* = 12 samples from GSE155698) and healthy normal pancreata (*n* = 5 donors from GSE229413) were performed using Wilcoxon rank-sum tests. Effect sizes were reported as Cohen’s d, computed as the difference in group means divided by the pooled standard deviation. The adjacent normal samples from GSE155698 (*n* = 3) were retained as a secondary comparison to characterize field effects in peritumoral tissue.

### 2.7. Metabolic State Classification

Cells were classified into five discrete metabolic states. Primary classification used unified global quartile thresholds computed across all 32,227 cells regardless of condition: (1) glycolysis-dominant: top quartile for glycolysis and bottom quartile for OXPHOS; (2) OXPHOS-dominant: top quartile for OXPHOS and bottom quartile for glycolysis; (3) Multi-pathway-high: top quartile for both glycolysis and OXPHOS; (4) All-low: bottom quartile for both pathways; and (5) Intermediate/Mixed: all remaining cells. Condition-specific quartile thresholds were also applied as a sensitivity analysis. The global distribution of cells across metabolic states was compared between tumor and normal conditions using chi-square tests of independence with Benjamini–Hochberg correction.

### 2.8. Cell-Type-Resolved MRI Analysis

To determine whether the global MRI difference between tumor and normal compartments is driven by specific cell lineages, MRI values were compared between tumor-derived and normal-derived cells within each annotated cell type separately. Eight major lineages were analyzed: malignant/epithelial cells (KRT19+/EPCAM+), ductal cells, cancer-associated fibroblasts (CAFs), macrophages, CD8+ T cells, B cells, NK cells, and acinar cells. Within-lineage comparisons were performed using Wilcoxon rank-sum tests with Benjamini–Hochberg FDR correction. Sample-level mean overlays (per-sample mean MRI) were added to figures to enable visualization of inter-sample variability alongside cell-level distributions.

### 2.9. Inter-Pathway Correlation Analysis

Pairwise Pearson correlation coefficients among glycolysis, OXPHOS, and hypoxia pathway scores were computed separately for tumor-derived and normal-derived cells. Differences in correlation strength between conditions were assessed using Fisher’s Z-transformation, which converts Pearson r values to normally distributed Z scores. Three pairwise comparisons were performed: glycolysis–hypoxia, glycolysis–OXPHOS, and OXPHOS–hypoxia. All Fisher’s Z-transformation *p*-values were subjected to Benjamini–Hochberg FDR correction.

### 2.10. Survival Analysis in TCGA-PAAD

Bulk RNA-seq expression data and clinical annotations for 183 patients with pancreatic adenocarcinoma were obtained from The Cancer Genome Atlas (TCGA-PAAD) cohort via the NCI Genomic Data Commons portal using TCGAbiolinks (v2.30.0). A Warburg Index was computed per patient as the difference between mean log-transformed (log1p TPM) glycolysis gene expression (LDHA, PKM, ENO1, GAPDH, SLC2A1, HK2, PFKL, and ALDOA) and mean log-transformed OXPHOS gene expression (COX5A, UQCRC1, ATP5F1A, SDHB, NDUFB8, NDUFS1, and SDHA). Agreement between FPKM and TPM normalizations was confirmed by Pearson correlation (r = 0.999). Patients with available survival data (*n* = 164) were stratified into Warburg-High and Warburg-Low groups (*n* = 82 per group) using the median Warburg Index as the split threshold. Overall survival was compared using Kaplan–Meier analysis and the log-rank test. Multivariate Cox proportional hazard regression was fitted in 135 patients with complete covariate data, adjusting for age and AJCC pathologic stage. Model performance was assessed by the concordance index (C-index).

### 2.11. Cross-Cohort Validation

To assess the generalizability of MRI-based metabolic characterization, all primary analyses were independently replicated in a second publicly available PDAC scRNA-seq dataset (GEO accession GSE205013; Werba et al., 2023 [20]). This dataset comprises single-cell transcriptional profiles from 27 PDAC patients, including treatment-naive and chemotherapy-treated samples from both primary and metastatic sites. Only treatment-naive primary PDAC samples were retained (*n* = 17 samples; 34,000 cells after quality control and subsampling to 2000 cells per sample; set.seed (42)). Subsampling stability was assessed using three independent random seeds (42, 123, and 456); the coefficient of variation in mean MRI across seeds was 0.12%.

### 2.12. Drug-Sensitivity Analysis

Drug sensitivity data were obtained from the Genomics of Drug Sensitivity in Cancer database (GDSC2, release 8.5, October 2023 [22,23]). Sensitivity profiles for 30 PDAC cell lines (TCGA_DESC = ‘PAAD’) and 287 compounds were extracted. Drug sensitivity was quantified using the standardized Z-score of the natural log-transformed IC50 (LN_IC50), where negative Z-scores indicate relative sensitivity relative to the pan-cancer distribution. Pathway-level sensitivity was summarized by computing mean Z-scores per drug across available PDAC cell lines (restricted to compounds tested in ≥10 cell lines). The association between ERK MAPK and metabolic drug sensitivity was assessed using Pearson correlation, Spearman rank correlation, and robust regression (MASS::rlm, M-estimation with Huber weighting). KRAS mutation status was annotated for each PDAC cell line based on the published literature, and ERK–metabolic sensitivity correlations were computed separately within KRAS-mutant and KRAS-wild-type subgroups.

### 2.13. Protein–Protein Interaction Network Analysis

A protein–protein interaction network was constructed for the 23 unique metabolic genes comprising the glycolysis, OXPHOS, and hypoxia pathway panels. The 23 unique genes reflect overlapping membership across compact and extended pathway panels: the three compact sets contain 15 genes in total (5 per pathway), and the extended sets introduce additional genes per pathway; across both compact and extended sets combined, 23 unique genes were retained after removing cross-panel duplicates introduced by the extended gene sets. Edges were defined based on known functional associations from the STRING database (v11.5, confidence score threshold > 400 [24]), using the STRINGdb R package (v2.14.0). Network visualization and topology analysis were performed using the igraph package (v1.6.0) [25] in R. Hub nodes were identified as genes with the highest degree centrality across modules.

### 2.14. Tumor Microenvironment Deconvolution

Cell-type deconvolution was performed using the EPIC algorithm (v1.1.7 [26]) on TPM-normalized bulk RNA-seq expression matrices from 183 TCGA-PAAD samples. EPIC estimates proportions of B cells, cancer-associated fibroblasts (CAFs), CD4+ T cells, CD8+ T cells, endothelial cells, macrophages, NK cells, and other cells using the default reference matrix. Deconvolved cell fractions were compared between Warburg-High (*n* = 82) and Warburg-Low (*n* = 82) patient groups using Wilcoxon rank-sum tests with Benjamini–Hochberg FDR correction. To address tumor purity confounding, partial Pearson correlation was computed between the Warburg Index and CAF fraction, controlling for tumor purity (proxied by the otherCells fraction from EPIC). Partial correlations were computed using the ppcor R package (v1.1).

### 2.15. Statistical Analysis and Multiple Testing Correction

All primary Wilcoxon rank-sum tests, chi-square tests of independence, and Fisher’s Z-transformation tests were subjected to Benjamini–Hochberg FDR correction. Adjusted *p*-values (*p*.adj) are reported throughout. A significance threshold of α = 0.05 was applied after correction. Wilcoxon rank-sum tests were selected for MRI comparisons given the right-skewed distributions of MRI values. Statistical analyses were performed in R (version 4.4.1). Random seed was fixed at set.seed (42) for all stochastic operations.

## 3. Results

### 3.1. Sample-Level Pseudobulk Analysis Validates MRI Difference Against Healthy Pancreas

**Figure 1:**
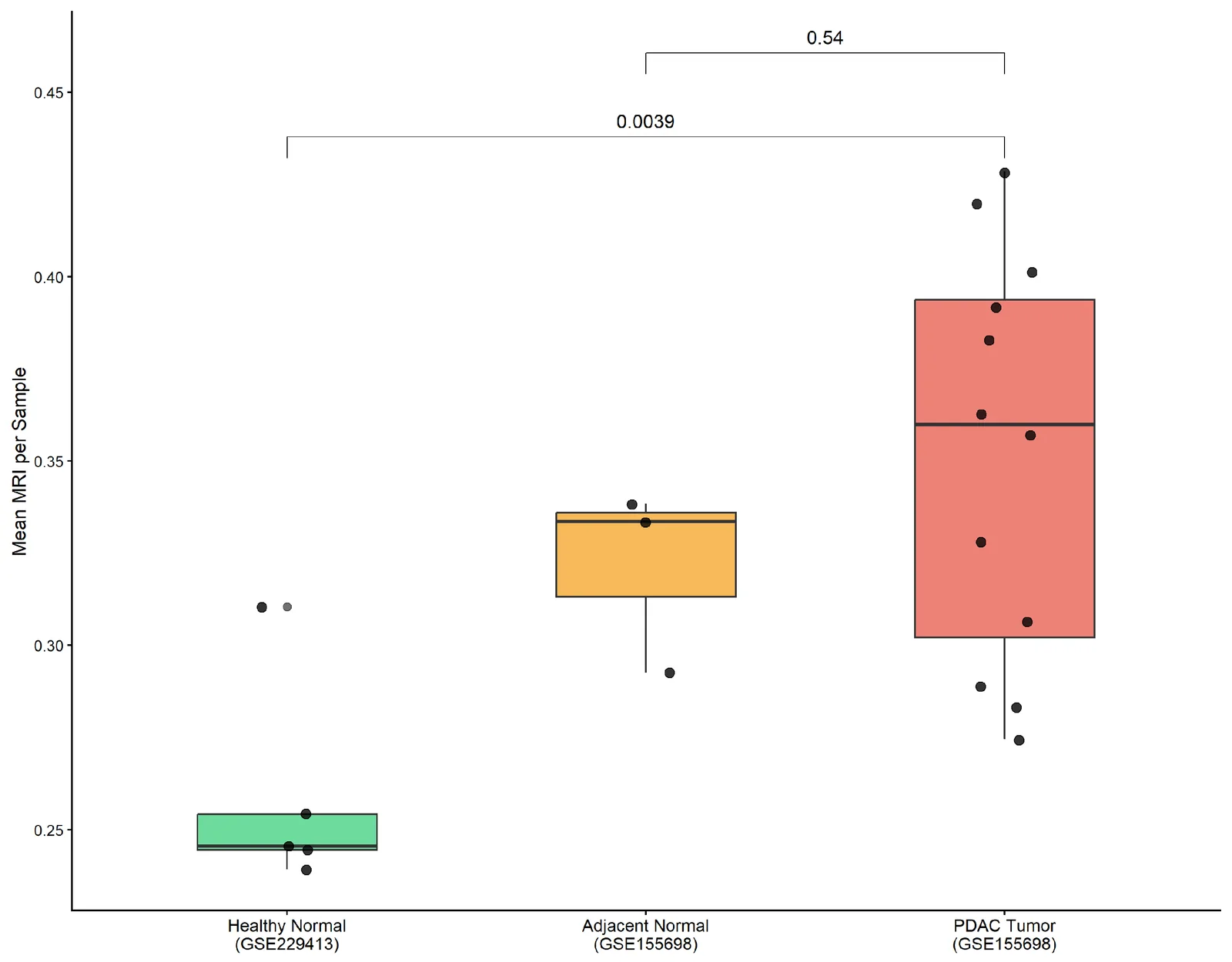
Sample-level MRI: pseudobulk analysis. Box plots showing mean MRI per sample across healthy normal pancreata (GSE229413; *n* = 5, green), adjacent normal (GSE155698; *n* = 3, orange), and PDAC tumor (GSE155698; *n* = 12, red). Individual dots represent per-sample mean MRI values. Pseudobulk comparison: Wilcoxon *p* = 0.0039, Cohen’s d = 1.437. MRI: Metabolic Rheostat Index.

To address pseudoreplication concerns arising from treating individual cells as independent observations, we performed a pseudobulk sample-level analysis aggregating per-cell MRI values to per-sample means. We incorporated an independent healthy pancreas scRNA-seq dataset (GSE229413; *n* = 5 donors, 32,627 cells after QC) as a biologically appropriate normal reference with a sufficient sample size. Pseudobulk comparison between PDAC tumor samples (*n* = 12, GSE155698) and healthy normal donors (*n* = 5, GSE229413) demonstrated a significantly higher MRI in PDAC tumor relative to healthy pancreata (mean: 0.352 vs. 0.259; Wilcoxon *p* = 0.0039; Cohen’s d = 1.437; Figure 1). This large effect size confirms that the MRI difference is not a statistical artifact of large cell numbers but reflects a genuine biological difference in inter-pathway metabolic organization. Adjacent normal samples from GSE155698 showed intermediate MRI values (mean = 0.322), consistent with microenvironmental field effects in peritumoral tissue (*p* = 0.54 vs. tumor, NS).

Single-cell RNA sequencing analysis of 32,227 cells from GSE155698 (24,515 tumor-derived, 7712 normal) revealed clear spatial partitioning of tumor and normal cells in UMAP space, with 37 transcriptionally distinct clusters annotated into 12 major lineages using canonical marker genes (Supplementary Figures S1 and S2). Overlay of metabolic states confirmed that no state was restricted to a single cluster or tissue compartment, indicating that metabolic phenotype and transcriptional identity are not rigidly co-determined.

**Figure 2:**
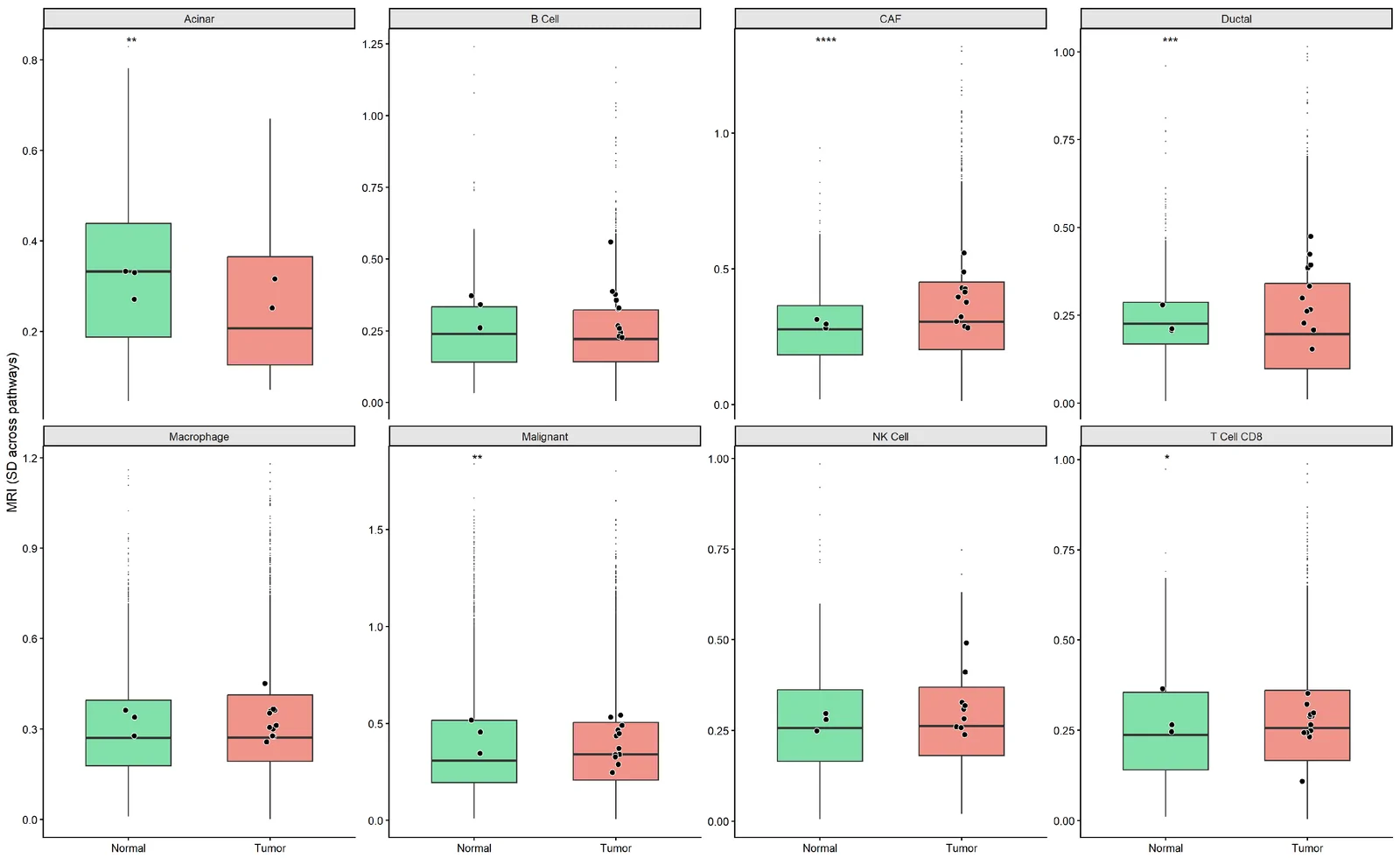
Cell-type-resolved MRI analysis in PDAC. Box plots of MRI values stratified by cell type (facets) and condition (green, normal; red, tumor). Black dots = per-sample means. Eight lineages shown. Significance: BH-adjusted Wilcoxon rank-sum test. * *p*.adj < 0.05, ** *p*.adj < 0.01, *** *p*.adj < 0.001, **** *p*.adj < 0.0001. CAF: cancer-associated fibroblast; MRI: Metabolic Rheostat Index.

To determine whether the global MRI difference is driven by specific lineages or represents a distributed phenomenon, we performed a cell-type-resolved MRI analysis across eight major lineages. CAFs showed the strongest tumor > normal difference (mean: 0.359 vs. 0.285; Wilcoxon *p*.adj = 3.5 × 10^−7^), consistent with microenvironment-driven metabolic remodeling of stromal cells. Malignant/epithelial cells (KRT19+/EPCAM+) showed a normal > tumor difference (mean: 0.393 vs. 0.379; *p*.adj = 0.009), the opposite direction from the tissue-level pseudobulk increase reported above. Ductal cells showed a tumor > normal difference (*p*.adj = 0.0004), and acinar cells showed a normal > tumor difference (*p*.adj = 0.011). Macrophages, B cells, and NK cells did not differ significantly (*p*.adj > 0.05; Figure 2). These lineage-specific patterns indicate that the tissue-level and sample-level increase in MRI is driven predominantly by CAFs and ductal cells rather than by the malignant epithelial compartment, which itself shows a modest decrease, demonstrating that compositional shifts, not uniform cell-intrinsic reprogramming, underlie the whole-tissue signal cross within the tumor ecosystem.

### 3.2. Unified Global Threshold Classification Reveals Condition-Specific Metabolic State Shifts

**Figure 3:**
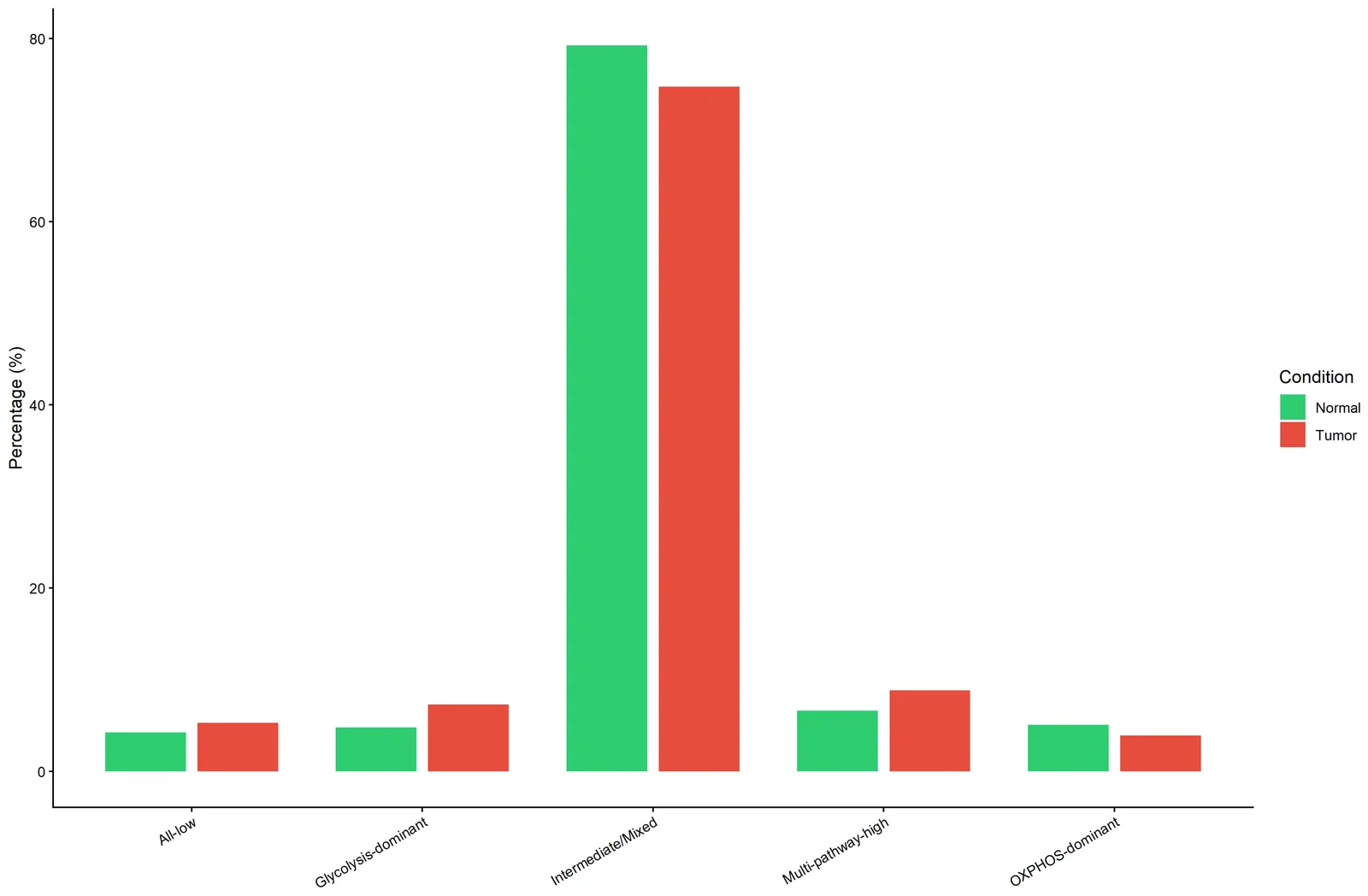
Metabolic state distribution: unified global threshold. Grouped bar chart showing proportion of cells in five metabolic states (All-low, glycolysis-dominant, Intermediate/Mixed, Multi-pathway-high, and OXPHOS-dominant) stratified by condition (green, normal; red, tumor). Unified global quartile thresholds were applied across all 32,227 cells. Intermediate/Mixed: 79.3% normal vs. 74.7% tumor. Chi-square *p*.adj < 0.001 (Benjamini–Hochberg correction).

The Metabolic Rheostat Index (MRI) was defined as the standard deviation across per-cell glycolysis, OXPHOS, and hypoxia pathway scores. A high MRI reflects metabolic polarization toward a dominant pathway; a low MRI reflects uniform cross-pathway engagement. To address the structural constraint imposed by condition-specific quartile thresholds, which by definition equalize the Intermediate/Mixed proportion across conditions, metabolic state classification was repeated using unified global quartile thresholds computed across all 32,227 cells. Under this approach, Intermediate/Mixed comprised 79.3% of normal cells and 74.7% of tumor cells, no longer structurally constrained to be equal. Glycolysis-dominant cells were proportionally more abundant in tumor cells (7.3% vs. 4.8%), and OXPHOS-dominant cells were higher in normal cells (5.1% vs. 3.9%). A chi-square test confirmed a significant global difference in state proportions (*p*.adj < 0.001, Benjamini–Hochberg correction; Figure 3).

At the cell level, within the malignant epithelial compartment specifically, normal pancreatic epithelial cells exhibited a higher mean MRI than malignant epithelial cells (0.3918 vs. 0.3716; Wilcoxon *p* < 2 × 10^−16^); this compartment-specific comparison is distinct from the whole-tissue pseudobulk gradient reported above (Figure 1), as detailed under the cell-type-resolved analysis below. MRI robustness was confirmed by a Shannon entropy-based formulation (*p*.adj = 2.1 × 10^−13^; Supplementary Figure S3), and the gene-set-sensitivity analysis confirmed directional consistency with extended 14–15 gene sets (Supplementary Figure S4). Pathway scoring was additionally validated using MSigDB hallmark gene sets (200 genes) and UCell rank-based scoring; three of four method combinations showed directionally consistent results (tumor normal MRI).

**Figure 4:**
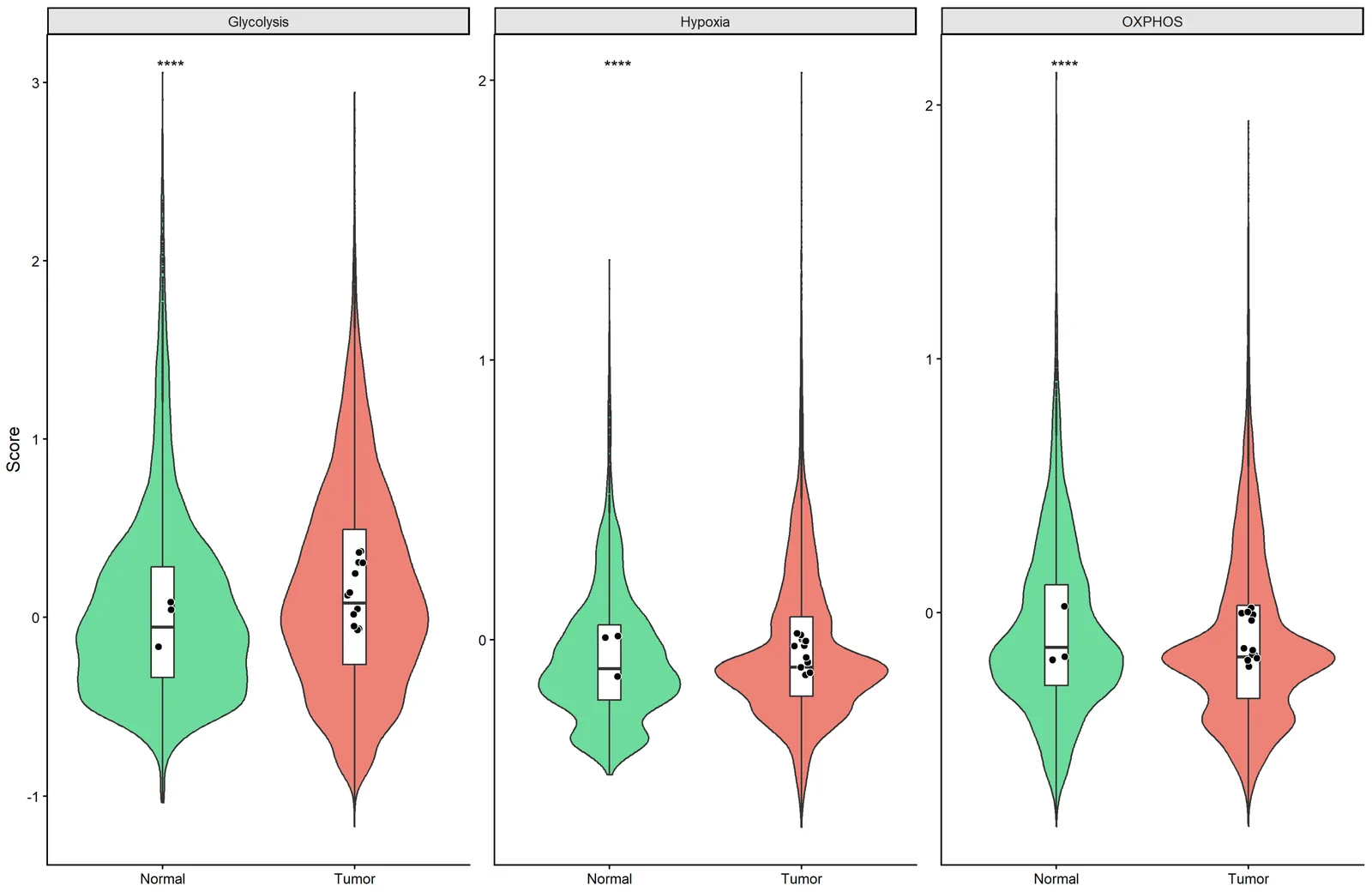
Metabolic pathway scores in PDAC: tumor vs. normal. Violin plots with overlaid box plots comparing per-cell pathway scores (glycolysis, OXPHOS, and hypoxia) between normal (green) and tumor (red) cells (*n* = 32,227 from GSE155698). Black dots = per-sample means. **** *p*.adj < 0.0001 (Benjamini-Hochberg corrected Wilcoxon rank-sum test).

Glycolysis scores were significantly elevated in tumor cells (*p*.adj < 2 × 10^−16^), OXPHOS scores were suppressed (*p*.adj < 2 × 10^−16^), and hypoxia scores were significantly reduced (*p*.adj = 9.1 × 10^−8^; Figure 4). Sample-level mean overlays confirmed these patterns were consistent across individual patients. The glycolysis–hypoxia inter-pathway correlation was significantly stronger in tumor cells (r = 0.121 vs. r = 0.019; Fisher Z, *p*.adj = 2.48 × 10^−8^), consistent with HIF-1α-mediated transcriptional co-activation. The glycolysis–OXPHOS correlation was significantly attenuated in tumor cells (r = 0.091 vs. r = 0.122; *p*.adj = 0.013), indicative of partial uncoupling of glycolytic and mitochondrial oxidative programs. The OXPHOS–hypoxia correlation did not differ significantly (*p*.adj = 0.052).

### 3.3. Warburg Index Associates with Overall Survival in TCGA-PAAD at Trend Level

**Figure 5:**
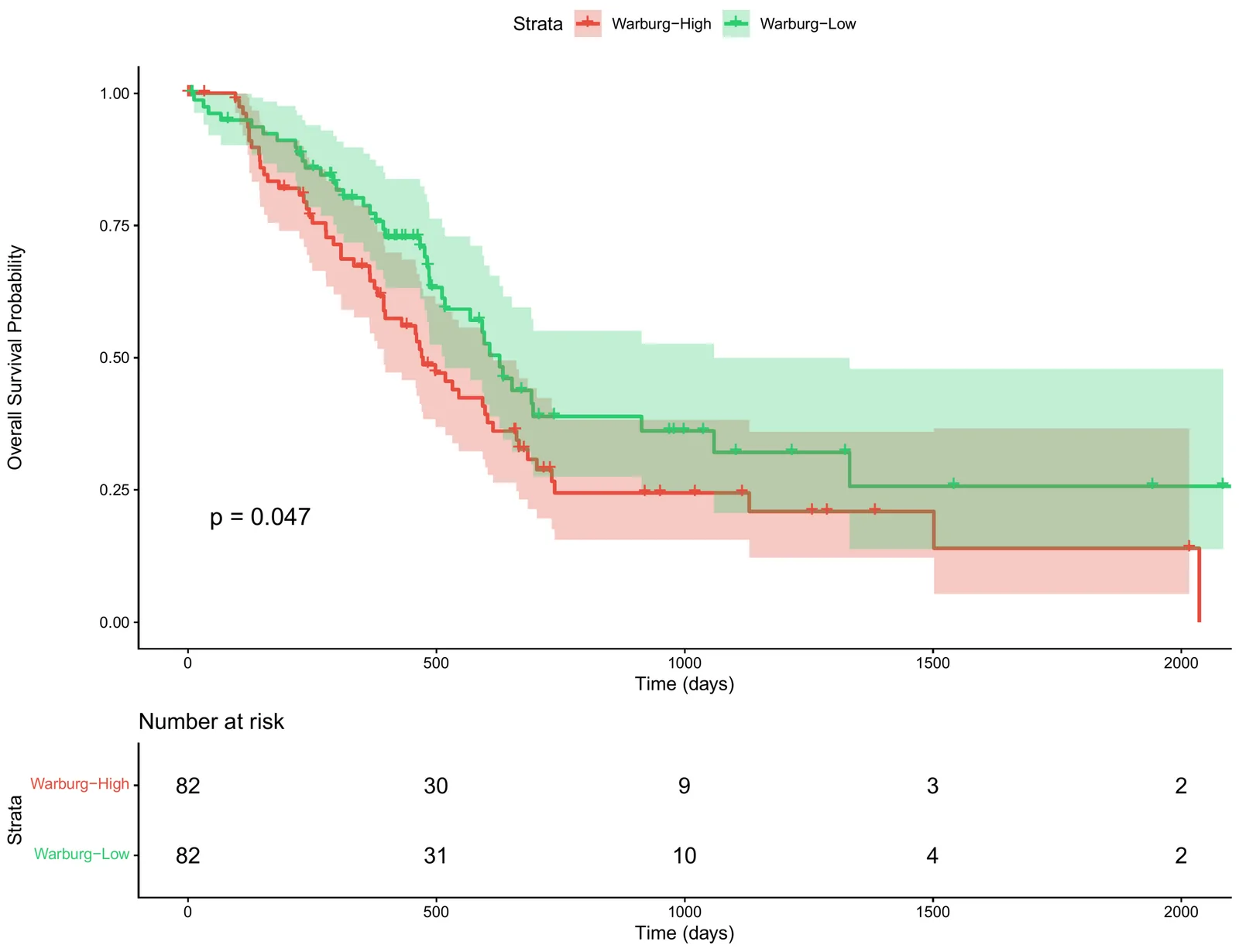
TCGA-PAAD: Warburg Index vs. overall survival. Kaplan–Meier curves for overall survival in 164 TCGA-PAAD patients stratified by Warburg Index (High vs. Low; *n* = 82 per group). Log-rank *p* = 0.047 (FPKM normalization); *p* = 0.051 (TPM normalization). Number-at-risk table shown below. Warburg Index TPM-FPKM correlation: r = 0.999.

A Warburg Index, defined as mean log-transformed glycolysis gene expression minus mean log-transformed OXPHOS gene expression, was computed for 164 TCGA-PAAD samples. Kaplan–Meier analysis demonstrated a trend toward shorter overall survival in Warburg-High patients (log-rank *p* = 0.047 [FPKM]; *p* = 0.051 [TPM]; Figure 5). FPKM and TPM normalizations yielded near-identical values (r = 0.999). Multivariate Cox proportional hazard regression (*n* = 135 patients with complete covariate data; 29 excluded due to missing age or stage) yielded a hazard ratio of 0.78 for Warburg-Low groups (95% CI: 0.48–1.30; *p* = 0.321; Supplementary Figure S5), with C-index = 0.56, indicating that the Warburg Index does not function as an independent prognostic marker after adjustment for clinical covariates.

**Figure 6:**
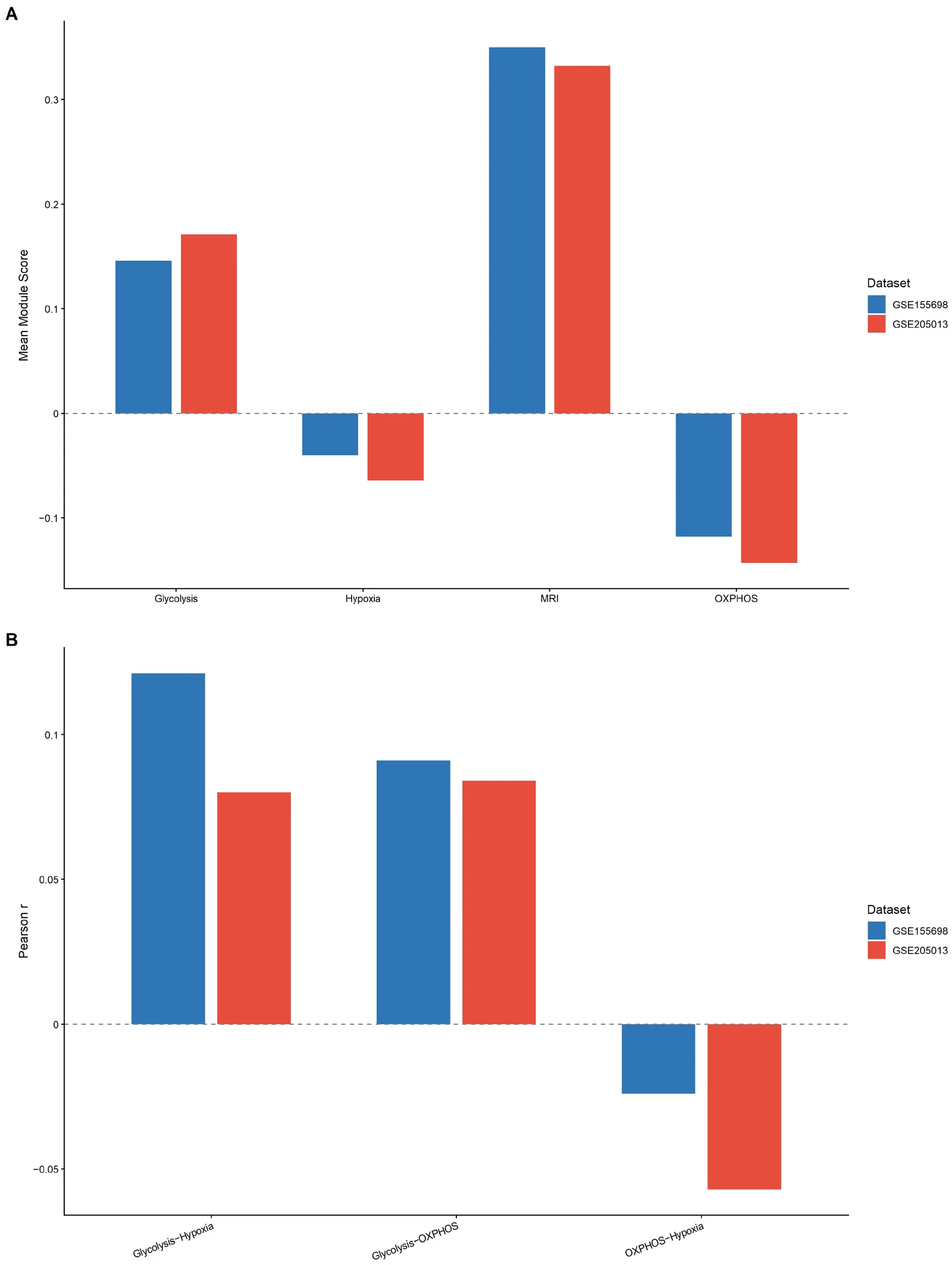
Cross-cohort validation: GSE155698 vs. GSE205013. (**A**) Mean module scores for Glycolysis, OXPHOS, Hypoxia, and MRI in GSE155698 (blue; *n* = 24,515 tumor cells) and GSE205013 (red; *n* = 34,000 cells). Dashed line indicates zero reference. (**B**) Pairwise inter-pathway Pearson correlations (Glycolysis−OXPHOS, Glycolysis−Hypoxia, OXPHOS−Hypoxia) in both cohorts. Dashed line indicates zero reference. Directional consistency across independent cohorts confirms reproducibility of the MRI framework. CV = 0.12% across three subsampling seeds.

To assess generalizability, all primary analyses were replicated in an independent PDAC scRNA-seq dataset (GSE205013; Werba et al., 2023 [20]). From this dataset, only treatment-naive primary PDAC samples were retained (*n* = 17 samples; 34,000 cells after QC and subsampling). Mean pathway scores were directionally consistent across both cohorts: glycolysis elevated (GSE155698: 0.146; GSE205013: 0.171), OXPHOS suppressed (−0.118 vs. −0.143), hypoxia negative (−0.040 vs. −0.064), and MRI concordant (0.350 vs. 0.332; Figure 6A). Inter-pathway correlations were also directionally preserved: glycolysis–OXPHOS remained weak (r = 0.091 vs. r = 0.084), glycolysis–hypoxia similarly modest (r = 0.121 vs. r = 0.080), and OXPHOS–hypoxia near-zero and negative in both cohorts (r = −0.024 vs. r = −0.057; Figure 6B). Subsampling stability analysis using three random seeds (42, 123, and 456) yielded an MRI coefficient of variation of 0.12%, confirming exceptional stability across subsampling realizations. We note that GSE205013 contains only treatment-naive primary tumor samples and lacks a matched adjacent-normal or healthy-pancreas control; this cross-cohort comparison therefore validates the reproducibility of PDAC-intrinsic pathway-score distributions and inter-pathway correlations and does not itself provide independent validation of the tumor-versus-normal MRI difference, which rests on the pseudobulk analysis incorporating GSE229413 described above.

### 3.4. ERK MAPK Inhibitors Represent the Primary Pharmacological Vulnerability in PDAC

**Figure 7:**
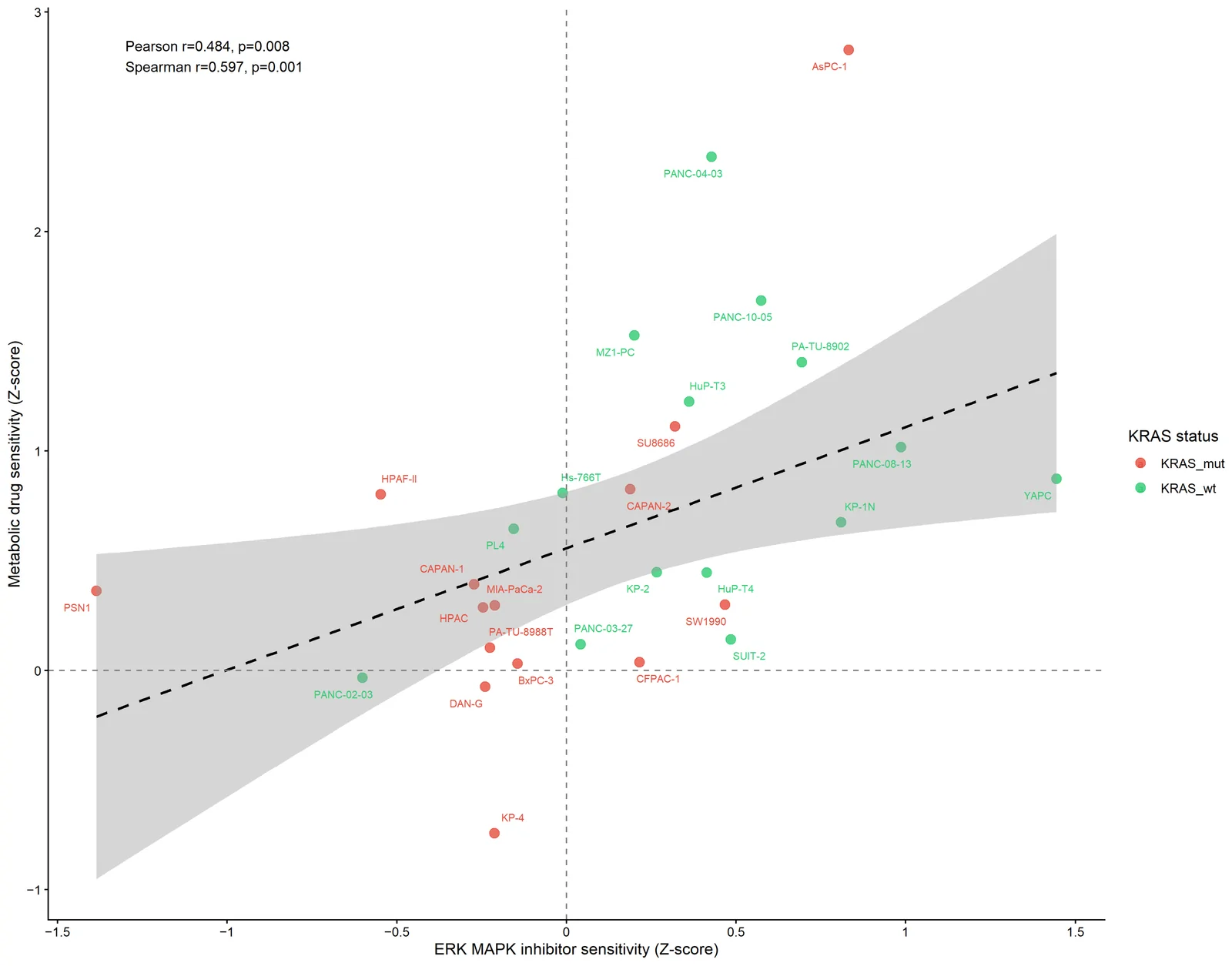
PDAC drug sensitivity landscape (GDSC2). Scatter plot of ERK MAPK vs. metabolic drug sensitivity (Z-scores) for 29 PDAC cell lines. Points colored by KRAS mutation status (red = KRAS mutant, green = KRAS wild-type). Dashed line: robust regression fit (MASS::rlm); gray shading indicates 95% confidence interval. Vertical and horizontal gray dashed lines indicate zero reference. Pearson r = 0.484 (*p* = 0.008); Spearman r = 0.597 (*p* = 0.0008); Robust regression coefficient = 0.553 (t = 2.71). GDSC2: Genomics of Drug Sensitivity in Cancer version 2.

Drug sensitivity profiles for 30 PDAC cell lines across 287 compounds were analyzed using GDSC2 data [22,23]. ERK MAPK pathway inhibitors showed the greatest sensitivity: PD0325901 (Z = −0.344), Selumetinib (Z= −0.262), Refametinib (Z = −0.222), SCH772984 (Z = −0.155), and Trametinib (Z= −0.098). ERK MAPK sensitivity correlated significantly with metabolic drug sensitivity using robust regression (MASS::rlm coefficient 0.553, t = 2.71), Spearman rank correlation (r = 0.597, *p* = 0.0008), and Pearson correlation (r = 0.484, *p* = 0.008; Figure 7). KRAS-mutant cell lines (*n* = 14) showed directionally consistent co-vulnerability (r = 0.485, *p* = 0.079), with the non-significant result attributable to the limited sample size. Cell lines PSN1 and KP-4 showed the greatest combined sensitivity; KP-2, SUIT-2, and PL4 displayed relative resistance.

### 3.5. Warburg-High PDAC Tumors Exhibit an Immunosuppressive Microenvironment

**Figure 8:**
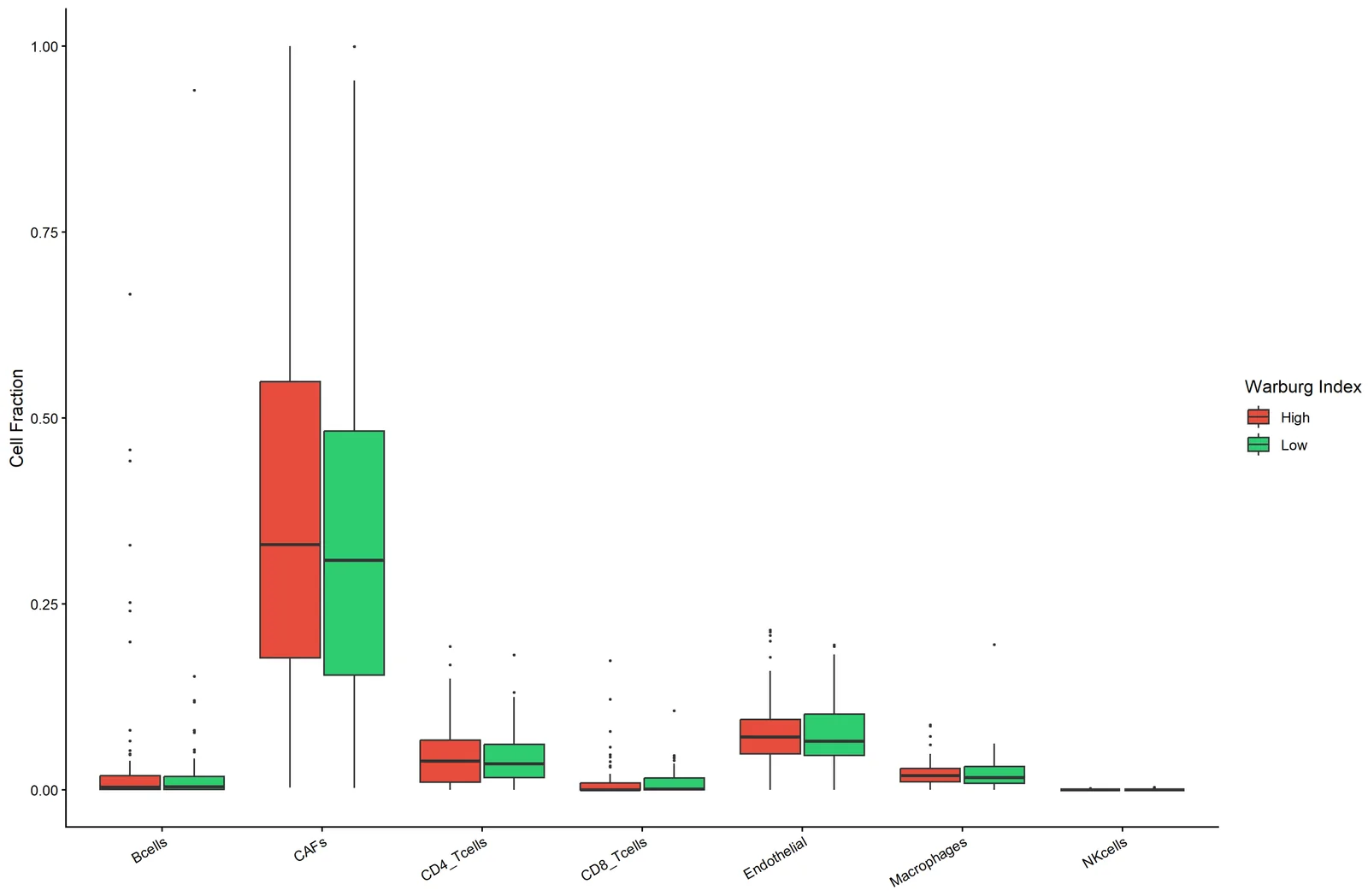
Tumor microenvironment composition by Warburg Index in TCGA-PAAD. Box plots of EPIC-deconvolved cell-type fractions stratified by Warburg Index group (High vs. Low; *n* = 82 per group). Horizontal dashed lines within boxes indicate group medians. Purity-adjusted partial r(CAF) = 0.312, *p* = 1.76 × 10^−5^. Significance: BH-adjusted Wilcoxon rank-sum test. *p*-values are expressed in scientific notation (e.g., 1.96 × 10^−6^). EPIC: Estimating the Proportion of Immune and Cancer cells; TCGA-PAAD: The Cancer Genome Atlas Pancreatic Adenocarcinoma.

EPIC deconvolution was applied to 183 TCGA-PAAD bulk RNA-seq samples [26]. Warburg-High tumors showed significantly higher CAF fractions (mean: 0.44 vs. 0.32; Wilcoxon *p*.adj = 0.005). To address tumor purity confounding, partial Pearson correlation was computed between the Warburg Index and CAF fraction, controlling for tumor purity (otherCells fraction as proxy). The purity-adjusted partial correlation remained significant (partial r = 0.312, *p* = 1.76 × 10^−5^), demonstrating that the CAF association is not attributable to differences in tumor cellularity. B cell infiltration was markedly reduced in Warburg-High tumors (mean: 0.008 vs. 0.052; *p*.adj = 1.6 × 10^−5^), NK cell fraction was lower (*p*.adj = 0.003), and CD4+ T cell abundance was reduced (mean: 0.035 vs. 0.052; *p*.adj = 0.027; Figure 8).

### 3.6. Metabolic Gene Interaction Network Identifies HIF1A as Inter-Pathway Hub

**Figure 9:**
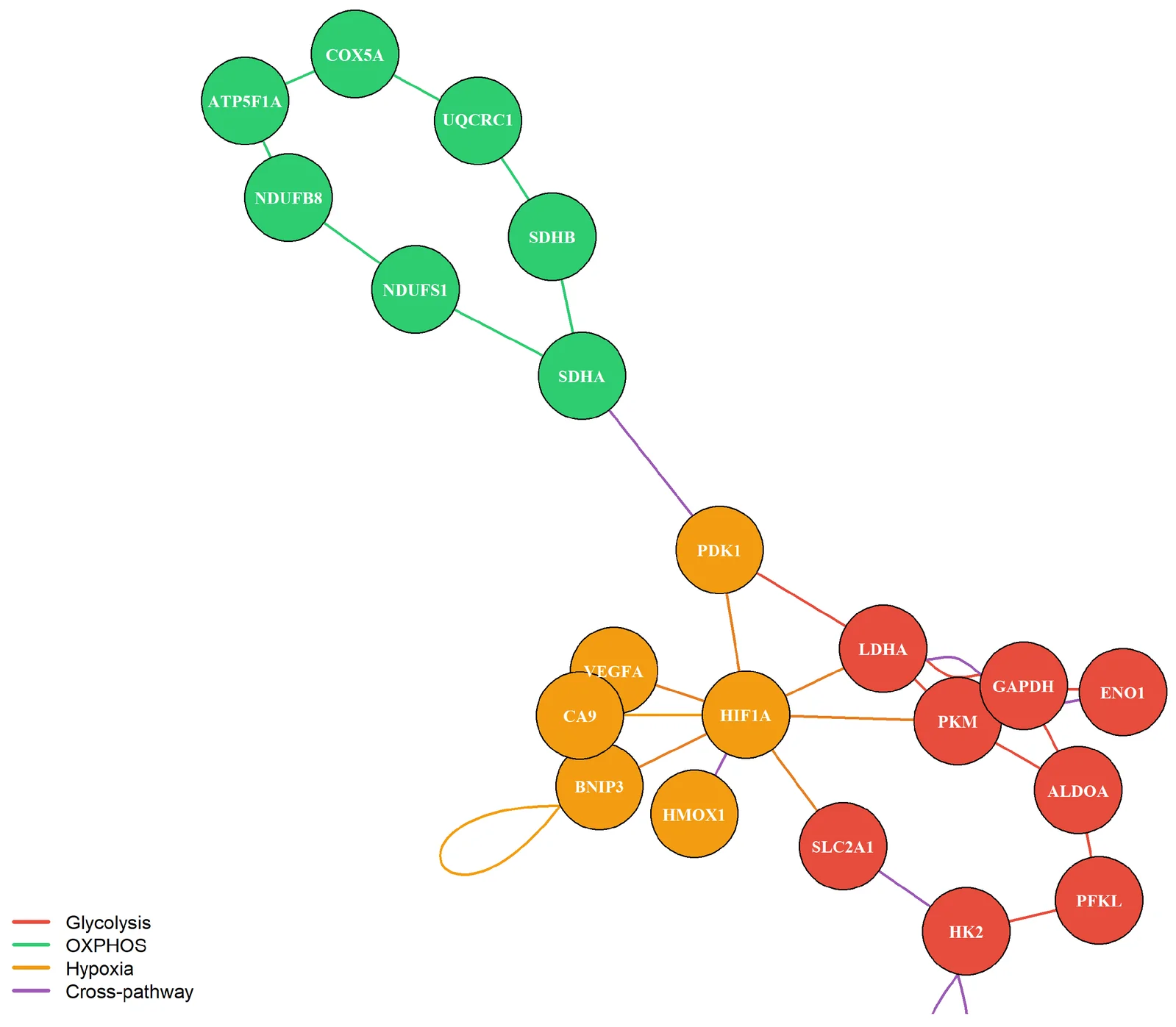
Metabolic gene protein–protein interaction network. Nodes colored by pathway: red (glycolysis), green (OXPHOS), and orange (hypoxia). Edge colors indicate interaction type: within-glycolysis (red), within-OXPHOS (green), within-hypoxia (orange), and cross-pathway (purple). HIF1A is the principal inter-module hub connecting glycolysis and hypoxia modules. PDK1 is the sole OXPHOS–hypoxia bridge. Network based on known functional associations from STRING database (v11.5, confidence score > 400).

To provide a mechanistic framework for the observed inter-pathway correlation patterns, a protein–protein interaction network was constructed for the 23 unique metabolic genes across the glycolysis, OXPHOS, and hypoxia panels, based on known functional associations from the STRING database (v11.5, confidence score > 400) [24,25]. Network analysis revealed three structurally distinct modules. The glycolysis module formed a densely interconnected cluster, and the OXPHOS module formed a structurally isolated subgraph with no direct edges to the glycolysis module (Figure 9), providing network-level mechanistic support for the weak glycolysis–OXPHOS correlation (r = 0.084–0.091) observed in both cohorts.

HIF1A emerged as the principal inter-module hub, bridging the hypoxia and glycolysis modules through known functional associations with LDHA, PKM, SLC2A1, PDK1, BNIP3, and VEGFA. This network topology is consistent with the significantly stronger glycolysis–hypoxia correlation in tumor cells (r = 0.121 vs. r = 0.019; *p*.adj = 2.48 × 10^−8^), although it does not independently demonstrate this relationship. The mechanistic basis for this correlation is supported by the prior experimental literature on HIF-1α-mediated transcriptional co-activation rather than network topology per se. PDK1 served as the sole cross-pathway bridge between the OXPHOS and hypoxia modules, consistent with PDK1-mediated suppression of pyruvate entry into the mitochondrial matrix.

## 4. Discussion

This study reveals a nuanced result when examined through the lens of adjacent normal tissue: metabolic divergence at the cell level appears more pronounced in adjacent normal pancreatic cells than in tumor cells. However, the pseudobulk sample-level analysis incorporating an independent healthy pancreas reference (GSE229413; *n* = 5 donors) revealed a more complete picture, in which MRI values form a gradient: healthy normal pancreas (mean = 0.259) < adjacent normal tissue (mean = 0.322) < PDAC tumor (mean = 0.352; Wilcoxon *p* = 0.0039; Cohen’s d = 1.437). This gradient is consistent with progressive metabolic polarization during malignant transformation and confirms that the MRI difference reflects a genuine biological phenomenon rather than a statistical artifact of large cell numbers. The intermediate MRI values in adjacent normal tissue are consistent with microenvironmental field effects in peritumoral tissue, where cells experience partial metabolic pressure from the developing tumor but have not yet undergone full malignant reprogramming [27].

The MRI captures the degree to which a cell’s metabolic programs are unevenly engaged, that is, the extent to which one pathway is markedly elevated while others are suppressed. A high MRI reflects metabolic polarization; a low MRI reflects metabolic uniformity, irrespective of absolute metabolic rate. Our cell-type-resolved analysis (Figure 2) bears directly on this point: glycolysis-dominant cells carry the highest MRI of any metabolic state in both tumor and normal compartments, because by definition a glycolysis-dominant cell has a large spread between its high glycolysis score and its suppressed OXPHOS and hypoxia scores. The MRI is therefore not a measure of metabolic flexibility in the sense of switching capacity; it is a measure of how unequally a cell distributes activity across its metabolic programs at any given moment. Critically, the cell-type-resolved analysis demonstrated that this pattern is not driven by a single lineage: CAFs showed the strongest tumor > normal difference (*p*.adj = 3.5 × 10^−7^), malignant/epithelial cells recapitulated the normal > tumor direction (*p*.adj = 0.009), and acinar cells showed a normal > tumor pattern (*p*.adj = 0.011), indicating a distributed metabolic reorganization across the tumor ecosystem.

Malignant/epithelial cells specifically carry a higher mean MRI than normal epithelium when compared at the individual-cell level (0.3918 vs. 0.3716; *p* < 2 × 10^−16^), indicating that in isolation, the malignant epithelial compartment achieves more coordinated pathway engagement than normal ductal/acinar epithelium. This cell-intrinsic pattern is compositionally distinct from the sample-level, whole-tissue pseudobulk signal reported above, which aggregates all cell types present in a tissue sample and is dominated by the pronounced tumor > normal shift in cancer-associated fibroblasts (*p*.adj = 3.5 × 10^−7^) and ductal cells (*p*.adj = 0.0004); the net result is that whole PDAC tissue shows greater inter-pathway divergence than normal pancreata, even though the malignant epithelial compartment itself is, if anything, more metabolically coordinated than normal epithelium. Several features of the data require careful interpretation. At the cell level, the absolute effect size is modest (~0.02 units), and the statistical significance is substantially amplified by the large sample (*n* = 32,227 cells). The sample-level pseudobulk analysis (Cohen’s d = 1.437) provides the primary effect size estimate, as it appropriately treats each patient or donor as the independent unit of observation. The CV-based MRI formulation yielded a non-significant result (*p* = 0.23), attributed to the mathematical instability of the coefficient of variation near zero-mean data; the highly significant entropy-based formulation (*p*.adj = 2.1 × 10^−13^) and the UCell–compact scoring approach support the biological reality of the finding. Pathway scoring validation using MSigDB hallmark gene sets and UCell confirmed directional consistency in three of four method combinations, establishing metric-independent robustness of the MRI direction.

One candidate explanation is the convergent pressure of the PDAC microenvironment on individual cell-level metabolic states. The PDAC stroma, among the most desmoplastic in oncology, is chronically hypoxic, nutrient-depleted, mechanically stiff, and immunosuppressive, and it exerts powerful metabolic pressure on embedded cells [28]. Under sustained HIF-1α activation, driven by matrix stiffness via a CLIC1-ROS signaling axis in PDAC [11], both glycolytic and hypoxia-response programs are co-activated simultaneously in the same cells. This co-activation reduces the spread between glycolysis and hypoxia scores within each tumor cell, directly lowering within-cell SD, which is the pattern captured by the lower MRI in tumor cells. The intermediate MRI values observed in adjacent normal samples (mean = 0.322, between healthy normal, 0.259, and tumor, 0.352) are consistent with microenvironmental field effects in peritumoral tissue, where cells experience partial metabolic pressure from the developing tumor but have not yet undergone full malignant reprogramming.

The glycolysis–hypoxia inter-pathway correlation is significantly tighter in tumor cells (r = 0.121 vs. r = 0.019; *p*.adj = 2.48 × 10^−8^), supporting a model in which HIF-1α functions as a master transcriptional co-activator that simultaneously upregulates glycolytic enzymes and hypoxia-response genes [28]. This network-level interpretation is supported by our PPI analysis (Figure 9), in which HIF1A emerged as the principal inter-module hub bridging the glycolysis and hypoxia modules through known functional associations with LDHA, PKM, SLC2A1, PDK1, BNIP3, and VEGFA [24]. It is important to note that the PPI network topology is illustrative of known protein–protein associations rather than independent evidence of causal regulation; the mechanistic basis for the glycolysis–hypoxia correlation rests on the prior experimental literature rather than network topology per se. The concurrent loosening of the glycolysis–OXPHOS correlation (r = 0.091 vs. r = 0.122; *p*.adj = 0.013) is consistent with the Reverse Warburg Effect [9], wherein OXPHOS activity is increasingly sustained by stromal-derived catabolites rather than the cell’s own glycolytic output. PDK1, the sole cross-pathway bridge between the OXPHOS and hypoxia modules in the PPI network, provides a structural basis for this decoupling through its suppression of pyruvate entry into the mitochondrial matrix [13].

The reproducibility of the MRI framework across two independent PDAC scRNA-seq cohorts, GSE155698 (*n* = 32,227 cells) and GSE205013 (*n* = 34,000 cells from 17 treatment-naive primary PDAC tumors) [20], substantially strengthens the generalizability of our findings. The directional consistency of mean pathway scores, MRI values, and inter-pathway correlation coefficients across datasets generated by different laboratories confirms that the metabolic rheostat architecture is a robust feature of PDAC transcriptional biology. The concordance in glycolysis–OXPHOS correlation coefficients (r = 0.091 and r = 0.084, respectively) is particularly notable, as this near-independence of glycolytic and mitochondrial oxidative programs is the central mechanistic prediction of the rheostat model. Subsampling stability analysis (CV = 0.12% across three seeds) further confirms that the GSE205013 validation results are not sensitive to the choice of random seed.

These results fit within a growing body of single-cell pan-cancer evidence. A recent compendium integrating 296 tumor and normal samples across six cancer types, including PDAC, identified shared glycolysis upregulation across all malignant compartments alongside divergent regulation of the citric acid cycle [16]. Our cell-type-controlled analysis adds nuance: within the epithelial/ductal-like compartment (KRT19+/EPCAM+), normal-derived cells carry significantly higher glycolytic scores than their tumor-derived counterparts (*p*.adj = 0.0082), while OXPHOS scores do not differ (*p*.adj = 0.82), suggesting that the glycolytic signature of established PDAC is not uniformly elevated across all epithelial cells but is concentrated in a subset. Most recently, spatial transcriptomic mapping of glycolytic heterogeneity in primary human PDAC specimens revealed pronounced inter-patient and intra-tumor glycolytic variation correlating with clinical outcomes and differential sensitivity to metabolic inhibitors [29].

Relating the MRI findings to the established molecular subtypes of PDAC requires careful framing. Moffitt et al. identified two major tumor-intrinsic PDAC subtypes, classical and basal-like, with the basal-like subtype associated with worse prognosis and chemotherapy resistance [30]. A more recent comprehensive single-cell atlas resolved two distinct basal-like tumor populations with distinct spatial distributions and survival correlations [31], and single-cell profiling across disease stages demonstrated that transcriptional plasticity and drug response in PDAC are dynamically modulated by stage and microenvironment [32], consistent with the emergence of high-plasticity cell states described during epithelial tumor evolution more broadly [33]. Within our framework, one might be tempted to hypothesize that the glycolytic phenotype of basal-like cells would correspond to a low MRI, but this would be incorrect: as Figure 3 demonstrates, glycolysis-dominant cells carry the highest MRI of any metabolic state, because strong glycolytic commitment with concurrent OXPHOS and hypoxia suppression produces exactly the large cross-pathway spread that defines a high SD. The relationship between molecular subtypes and MRI scores is therefore likely more nuanced and will require datasets combining single-cell transcriptomic profiles with matched molecular subtype annotations.

The GDSC2 drug-sensitivity analysis [22,23] identifies ERK MAPK inhibitors as the primary pharmacological vulnerability in PDAC cell lines, consistent with the near-universal KRAS mutation in this disease and the established role of oncogenic KRAS4A in driving glycolytic flux through direct hexokinase 1 interaction [12]. The significant correlation between ERK MAPK and metabolic drug sensitivity across PDAC cell lines, confirmed by robust regression (coefficient = 0.553, t = 2.71), Spearman correlation (r = 0.597, *p* = 0.0008), and Pearson correlation (r = 0.484, *p* = 0.008), suggests that KRAS-driven metabolic reprogramming creates co-vulnerabilities that could be exploited therapeutically. We note that this analysis does not directly test whether MRI or single-cell metabolic coordination predicts drug sensitivity, since pathway scores and MRI were not computed in the same GDSC2 cell lines; we therefore present this drug sensitivity association as an exploratory, hypothesis-generating observation motivating future work that integrates single-cell profiling of PDAC cell lines with matched drug sensitivity data. The relatively modest sensitivity of PDAC lines to dedicated metabolic inhibitors as monotherapies is consistent with the metabolic flexibility conferred by the rheostat architecture: when one pathway is targeted, the semi-independent regulation of alternative programs may permit compensatory upregulation. This observation motivates combination strategies targeting both the ERK MAPK axis and specific metabolic nodes simultaneously, as argued by Fendt et al. [34].

The EPIC deconvolution analysis [26] reveals that the bulk Warburg Index captures not only the intrinsic tumor cell metabolic state but also systematic differences in tumor microenvironment composition. Tumor purity-adjusted partial correlation confirmed that the association between the Warburg Index and CAF infiltration (partial r = 0.312, *p* = 1.76 × 10^−5^) is not attributable to differences in tumor cellularity, establishing that the stromal enrichment in Warburg-High tumors reflects genuine microenvironmental remodeling rather than a confound of tumor purity. The association between a high Warburg Index and elevated CAF infiltration is consistent with the Reverse Warburg Effect [9], wherein glycolytic cancer cells metabolically reprogram adjacent stromal fibroblasts. The concurrent depletion of B cells (*p*.adj = 1.6 × 10^−5^), NK cells (*p*.adj = 0.003), and CD4+ T cells (*p*.adj = 0.027) in Warburg-High tumors suggests that glycolytic reprogramming and immune exclusion are mechanistically linked processes in PDAC, consistent with evidence that show that lactate secretion by glycolytic tumor cells directly suppresses NK cell cytotoxicity and impairs T cell effector function. Furthermore, tumor-associated macrophages in PDAC are reprogrammed by microbial tryptophan metabolites acting through the aryl hydrocarbon receptor, adopting immunosuppressive configurations that suppress CD8+ T cell activity [35].

The survival analysis in TCGA-PAAD is instructive about what bulk-tissue metabolic indices can and cannot show. The Warburg Index showed a trend toward worse overall survival that was directionally consistent across two normalization methods (r = 0.999) but was not sustained in multivariate Cox regression (C-index = 0.56). This result is unsurprising in light of our single-cell and deconvolution findings: the bulk Warburg Index conflates intrinsic tumor cell metabolism with microenvironmental CAF and immune cell composition, making it a composite rather than a pure signal. Compressing 32,227 cells with heterogeneous metabolic profiles into a single scalar per patient destroys the structural information that single-cell resolution preserves, as argued by Faubert et al. [36]. Future efforts toward metabolic prognostication in PDAC should prioritize deconvolution-based approaches or patient-derived organoid models in which MRI-type indices can be computed at the individual cell level.

A potential limitation of the pseudobulk comparison between GSE155698 and GSE229413 is the presence of dataset and batch effects. These two datasets were generated by different laboratories, using different tissue processing protocols, patient populations, and sequencing runs. GSE155698 comprises tumor and adjacent normal pancreatic tissue from PDAC patients [19], whereas GSE229413 comprises donor pancreata obtained from brain-dead donors without known pancreatic disease [21]. Differences in tissue dissociation protocols, sequencing depth, and cell-type composition between these datasets could contribute to systematic differences in pathway scores independent of true biological differences. To mitigate this concern, pathway scores were computed using the same gene sets and normalization pipeline in both datasets, and MRI values were compared at the sample level using non-parametric statistics that are robust to distributional differences. Nonetheless, formal batch correction integrating both datasets was not performed, as the primary comparison of interest was between healthy donors and PDAC tumor samples rather than across the full integrated embedding. Future studies integrating multiple normal pancreas references with harmonization methods, such as Harmony or ComBat-seq, would strengthen the cross-dataset comparison.

Several methodological limitations apply. Despite cross-cohort validation in GSE205013 and the pseudobulk analysis incorporating GSE229413 healthy donors, both datasets are retrospective reanalyses of publicly available data, and prospective single-cell profiling with matched clinical outcomes is required for definitive prognostic validation. The five-gene pathway sets, while validated against extended alternatives and MSigDB hallmark gene sets, remain parsimonious approximations with moderate correlations for OXPHOS and hypoxia (r = 0.54 and r = 0.47–0.61). The MRI is bounded by its three-pathway panel; extension to include fatty acid oxidation, glutaminolysis, the pentose phosphate pathway, and one-carbon metabolism would yield a richer characterization of inter-pathway architecture. The modest absolute effect size of the MRI difference at the cell level (~0.02 units) should be foregrounded in any clinical or translational application; the sample-level Cohen’s d = 1.437 provides the more appropriate effect size estimate. The GDSC2 drug-sensitivity analysis is limited to 30 PDAC cell lines and does not directly connect the cell line metabolic state to MRI; future work integrating single-cell profiling of PDAC cell lines with drug sensitivity data would directly address this limitation. Malignant cell identity in the adjacent normal tissue was based on KRT19/EPCAM/MUC1 expression without copy number variation analysis; these cells are therefore referred to as epithelial/ductal-like rather than confirmed malignant throughout. A CV-based MRI formulation should be discouraged for sparse scRNA-seq data with near-zero mean pathway scores. As a single summary statistic, MRI cannot on its own distinguish genuine single-pathway dominance from uniformly low or uniformly high expression across all three modules, including effects driven by gene-set composition, cell-cycle state, or stress-response programs; we report the three individual pathway scores alongside MRI throughout to allow for these scenarios to be distinguished, and this ambiguity should be kept in mind whenever a single MRI value is interpreted in isolation. We have not cross-validated the EPIC-based tumor purity adjustment using an independent purity estimator, such as ESTIMATE, or an alternative deconvolution method, such as CIBERSORTx or quanTIseq. The protein–protein interaction network is illustrative of known functional associations and does not constitute regulon inference, for example via SCENIC or DoRothEA, nor spatial or experimental validation of HIF1A’s regulatory role. We have not formally regressed out the UMI count, detected-gene count, mitochondrial read percentage, cell-cycle phase, or stress-response gene expression from the pathway scores; covariate-adjusted scoring is a priority for future refinement. Given the limited number of compounds within individual drug classes in the GDSC2 dataset, a fully powered drug-class-specific analysis was not possible.

Despite these limitations, this study provides a validated single-cell framework for quantifying metabolic divergence across pathways in PDAC and demonstrates, with statistical robustness across an independent healthy-pancreas reference cohort, a pseudobulk sample-level analysis (Cohen’s d = 1.437), cell-type-resolved comparisons, and multiple scoring method validations, that PDAC tumor tissue displays greater inter-pathway divergence than the normal pancreatic tissue from which it arises, following a monotonic gradient from healthy normal to adjacent normal to tumor. This tissue-level polarization is driven predominantly by cancer-associated fibroblasts and ductal cells rather than by the malignant epithelial compartment itself, which, examined in isolation, shows a modest but significant reduction in inter-pathway divergence relative to normal epithelium. The selective rewiring of the glycolysis–hypoxia regulatory axis, together with this compartment-specific pattern, describes a tumor metabolic architecture in which stromal and compositional remodeling accounts for the population-level increase in metabolic divergence relative to normal tissue more than malignant cell-intrinsic reprogramming does. The healthy-adjacent-tumor gradient supports this pattern as a plausible feature of malignant transformation, although it requires validation in additional independent cohorts with matched normal tissue. The association of a high Warburg Index with immunosuppressive microenvironment remodeling, which remained significant after adjusting for tumor purity, together with ERK–metabolic co-vulnerabilities identified in PDAC cell lines, provides a multi-dimensional translational framework that extends the MRI concept from single-cell characterization toward therapeutic application. Whether the observed metabolic convergence reflects a microenvironmental constraint, a consequence of oncogenic transcriptional programs, or a selected adaptive state remains unresolved and represents an important direction for future mechanistic investigation.

## Data Availability

All raw single-cell RNA sequencing datasets analyzed in this study are publicly available from the Gene Expression Omnibus (GEO) repository: discovery cohort under accession number GSE155698 [19], validation cohort under accession number GSE205013 [20], and healthy pancreas reference dataset under accession number GSE229413 [21]. TCGA-PAAD bulk RNA-seq expression data and clinical annotations were obtained from the NCI Genomic Data Commons (GDC) portal and are publicly accessible. Drug sensitivity data were obtained from the Genomics of Drug Sensitivity in Cancer database (GDSC2, release 8.5; https://www.cancerrxgene.org). The Metabolic Rheostat Index (MRI) scores, processed datasets, R analysis scripts, and figures generated during this study are openly available in the Zenodo repository at https://zenodo.org/records/21727263 (accessed on 8 August 2026), which was updated during peer review to include the R scripts for the additional analyses performed in this revision (pseudobulk sample-level validation, cell-type-resolved MRI analysis, hallmark/UCell scoring validation, unified global threshold classification, GDSC2 robust regression, EPIC tumor purity-adjusted deconvolution, and subsampling stability analysis).

## Supplementary Materials

The following supporting information can be downloaded at: https://www.mdpi.com/article/10.3390/cimb48080820/s1.
