# Supplementary material for: The Metabolic Rheostat Index Uncovers Pathway-Level Coordination, Immunosuppressive Microenvironment Remodeling, and Therapeutic Co-Vulnerabilities in Pancreatic Ductal Adenocarcinoma
Source: Curr Issues Mol Biol. 2026 Aug 12;48(8):820. doi: 10.3390/cimb48080820 (PMC13510339; doi:10.3390/cimb48080820)
Supplement: Supplementary file 1 [file cimb-48-00820-s001.zip › cimb-4423206-supplementary.pdf]

SUPPLEMENTARY MATERİALS

The Metabolic Rheostat Index Uncovers Pathway-Level Coordination, Immunosuppressive Microenvironment Remodeling, and Therapeutic Co-Vulnerabilities in Pancreatic Ductal Adenocarcinoma

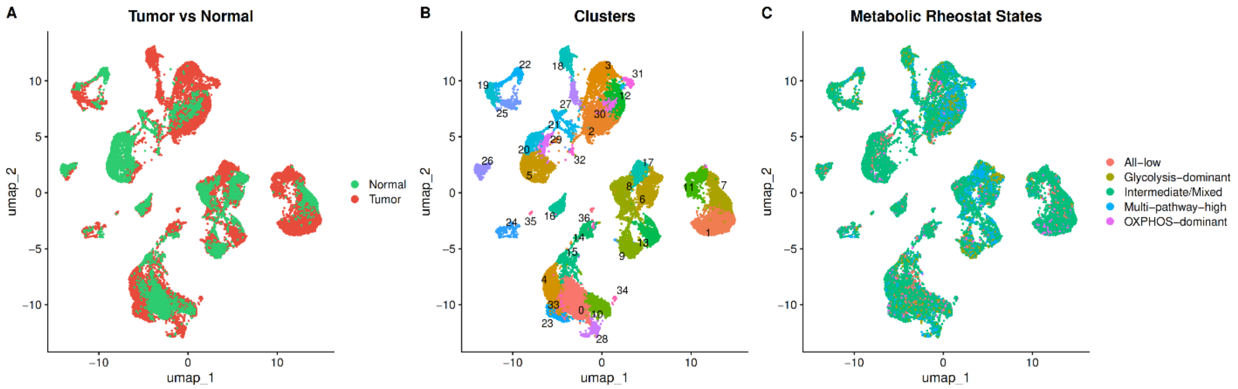

S1 Single-cell transcriptomic landscape of PAAD reveals metabolic state heterogeneity independent of cluster identity. UMAP projections of 32,227 cells (24,515 tumor-derived, 7,712 normal) from GSE155698. (A) Cells colored by tissue of origin (red, tumor; green, normal), showing broad spatial partitioning with partial overlap at transitional neighborhoods. (B) Unsupervised graph-based clustering resolving 37 transcriptionally distinct populations. (C) Overlay of five Metabolic Rheostat States (All-low, Glycolysis-dominant, Intermediate/Mixed, Multi-pathway-high, OXPHOS-dominant), demonstrating that metabolic phenotype is not restricted to any single cluster or tissue compartment.

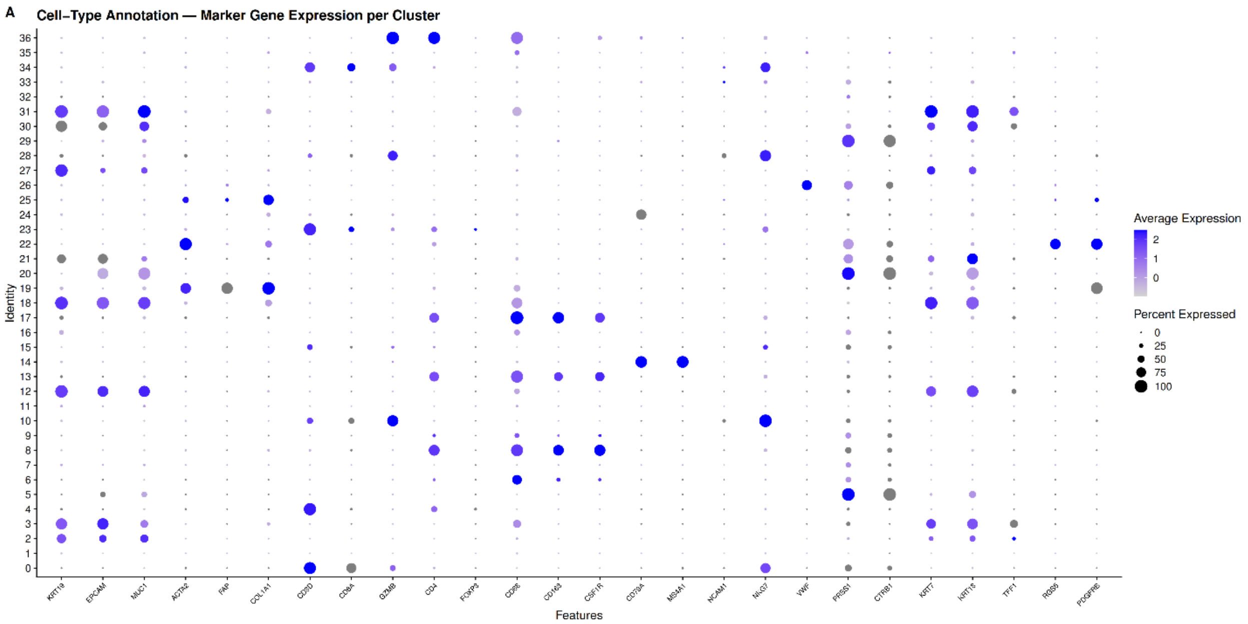

S2. Cell-type annotation of 37 clusters across twelve major lineages. Dot plot of canonical lineage marker expression across all clusters. Dot size indicates the fraction of cells expressing the marker; color intensity indicates mean scaled expression. Markers shown include KRT19, EPCAM, and MUC1 (malignant); KRT7, KRT18, and TFF1 (ductal); FAP, COL1A1, and ACTA2 (cancer-associated fibroblasts); CD68, CD163, and CSF1R (macrophages/myeloid); CD8A and GZMB (cytotoxic T cells); CD3D and CD4 (T helper cells); FOXP3 (Tregs); MS4A1 (B cells); NCAM1 and NKG7 (NK cells); VWF (endothelial); PRSS1 and CTRB1 (acinar); RGS5 and PDGFRB (stellate).

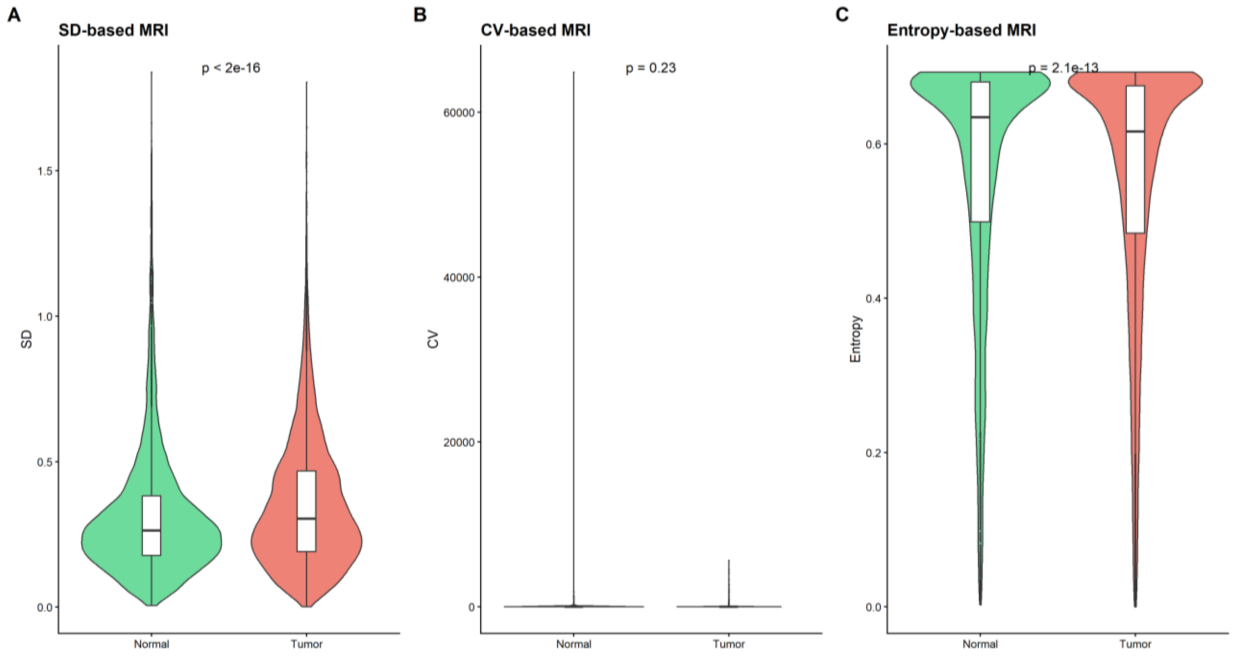

S3. Robustness of the Metabolic Rheostat Index Across Alternative Dispersion Metrics.

Violin plots comparing MRI distributions between normal (green) and tumor (red) cells (n=32,227 cells from GSE155698) using three dispersion formulations. (A) SD-based MRI: normal > tumor (Wilcoxon $p.adj<2\times10^{-16}$). (B) CV-based MRI: non-significant result ($p=0.23$), attributable to mathematical instability of the coefficient of variation when mean pathway scores approach zero, as evidenced by extreme outlier CV values exceeding 60,000 units. (C) Shannon entropy-based MRI, computed as $H=-\Sigma p\_i \log(p\_i)$ with scores transformed to a non-negative probability distribution; entropy-based MRI was defined as MRI_entropy=1−H_normalized such that higher values indicate greater inter-pathway divergence; normal > tumor ($p.adj=2.1\times10^{-13}$), confirming directional consistency with SD-based MRI. All p-values Benjamini-Hochberg corrected. MRI: Metabolic Rheostat Index; SD: standard deviation; CV: coefficient of variation.

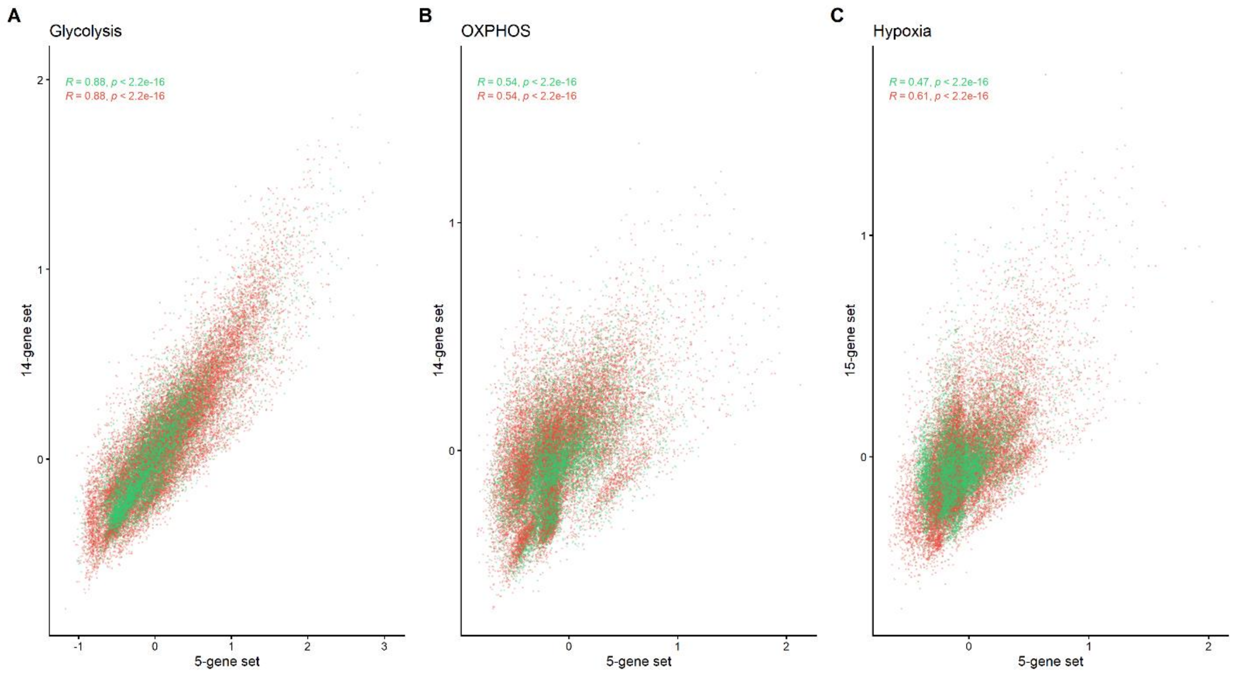

S4. Gene Set Composition Does Not Alter the Direction of Tumor-versus-Normal Metabolic Comparisons.

Scatter plots of per-cell pathway scores computed from compact five-gene sets (x-axis) versus extended gene sets (y-axis; 14-15 genes per pathway), with tumor (red) and normal (green) cells overlaid. (A) Glycolysis: compact (5 genes) versus extended (14 genes); Pearson r=0.88 ($p<2.2\times10^{-16}$). (B) OXPHOS: compact (5 genes) versus extended (14 genes); r=0.54. (C) Hypoxia: compact (5 genes) versus extended (15 genes); r=0.47-0.61. Crucially, the relative positions of tumor and normal cell clouds are preserved in directionality across all three panels regardless of gene set size, confirming that primary tumor-versus-normal comparisons are robust to gene set composition. Pathway scoring was additionally validated using MSigDB Hallmark gene sets (Glycolysis: 200 genes; OXPHOS: 201 genes; Hypoxia: 200 genes) and UCell rank-based scoring, with three of four method combinations showing directionally consistent results.

.

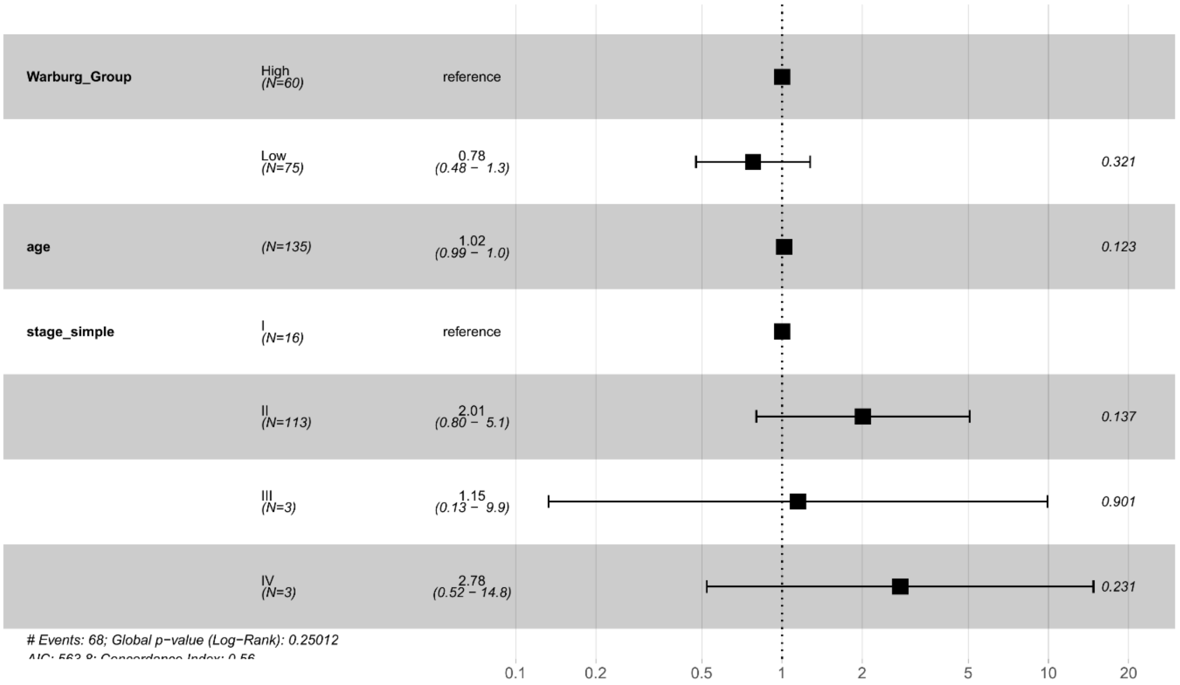

S5. Multivariate Cox Proportional Hazards Regression — Warburg Index Is Not an Independent Prognostic Marker in TCGA-PAAD

Forest plot from multivariate Cox proportional hazards regression adjusting for age and AJCC pathologic stage in 135 TCGA-PAAD patients with complete covariate data (29 patients excluded due to missing age or stage information). Warburg-Low group hazard ratio=0.78 (95% CI: 0.48-1.30; p=0.321), with the confidence interval crossing 1.0, indicating no independent prognostic value. Stage II showed the highest point estimate (HR=2.01; 95% CI: 0.80-5.10; p=0.137) relative to Stage I (n=16 reference group). Global model p=0.25; concordance index=0.56. These results indicate that the Warburg Index does not function as an independent prognostic marker after adjustment for clinical covariates in TCGA-PAAD. AJCC: American Joint Committee on Cancer; TCGA-PAAD: The Cancer Genome Atlas Pancreatic Adenocarcinoma.
